# Intuitive optics: what great apes infer from mirrors and shadows

**DOI:** 10.1007/s10071-018-1184-0

**Published:** 2018-05-02

**Authors:** Christoph J. Völter, Josep Call

**Affiliations:** 1 School of Psychology and Neuroscience, University of St Andrews, St Mary’s Quad, South Street, St Andrews, Fife, Scotland KY16 9JP UK; 2 Department of Developmental and Comparative Psychology, Max Planck Institute for Evolutionary Anthropology, Deutscher Platz 6, 04103 Leipzig, Germany

**Keywords:** Primate cognition, Causal cognition, Intuitive physics, Problem solving, Appearance-reality discrimination, Secondary representations

## Abstract

There is ongoing debate about the extent to which nonhuman animals, like humans, can go beyond first-order perceptual information to abstract structural information from their environment. To provide more empirical evidence regarding this question, we examined what type of information great apes (chimpanzees, bonobos, and orangutans) gain from optical effects such as shadows and mirror images. In an initial experiment, we investigated whether apes would use mirror images and shadows to locate hidden food. We found that all examined ape species used these cues to find the food. Follow-up experiments showed that apes neither confused these optical effects with the food rewards nor did they merely associate cues with food. First, naïve chimpanzees used the shadow of the hidden food to locate it but they did not learn within the same number of trials to use a perceptually similar rubber patch as indicator of the hidden food reward. Second, apes made use of the mirror images to estimate the distance of the hidden food from their own body. Depending on the distance, apes either pointed into the direction of the food or tried to access the hidden food directly. Third, apes showed some sensitivity to the geometrical relation between mirror orientation and mirrored objects when searching hidden food. Fourth, apes tended to interpret mirror images and pictures of these mirror images differently depending on their prior knowledge. Together, these findings suggest that apes are sensitive to the optical relation between mirror images and shadows and their physical referents.

**Electronic supplementary material:**

The online version of this article (10.1007/s10071-018-1184-0) contains supplementary material, which is available to authorized users.

## Introduction

A central debate in the field of comparative cognition is to what extent nonhuman animals go beyond the perceptual input to discriminate between appearance and reality (e.g., Carruthers 2008; Krachun et al. 2009) and to abstract higher-order relations (Penn et al. 2008). This is a pivotal question that transcends different areas of research including picture recognition (e.g., Fagot et al. 2010), object permanence (e.g., Jaakkola 2014), and causal cognition (e.g. Penn and Povinelli 2007). Suddendorf and Whiten (2001; following Perner 1991) proposed that secondary representations underlie each of these mental abilities in young children and nonhuman great apes (henceforth apes). Secondary representations, in contrast to primary representations that serve to directly model the current environment, involve a decoupling from the here and now. An important property of these secondary representations is that they enable organisms to integrate past knowledge and hypothetical scenarios with current perceptual information.

Appearance-reality (henceforth AR) discrimination tasks have been used in developmental and comparative studies to directly examine the ability to integrate current perceptual information with prior knowledge. In nonverbal AR discrimination tasks, individuals are usually presented first with all the relevant information (e.g., the true size of two different food items) that they need to make an informed decision (e.g., pick the larger of the two items). Thereafter, the appearance of the items is manipulated such that the preferred item now looks like the less-preferred one and vice versa. The appearance is altered, for example, by means of partial occlusion, magnifying and minimizing lenses, or colour filters. To pass these tests, subjects need to discount the currently misleading information and adhere to the previous information about the true item properties. Human children pass nonverbal AR tasks from about 2.5–3 years of age (Karg et al. 2014; Sapp et al. 2000). Apes have also mastered various versions of the AR discrimination task. Chimpanzees (*Pan troglodytes*) selected large grapes over small ones even when the large grapes at the time of choice appeared to be small (placed behind a minimizing lens) and the small ones appeared to be large (placed behind a magnifying lens; Krachun et al. 2009, 2016). Likewise, bonobos (*Pan paniscus*), chimpanzees, gorillas (*Gorilla gorilla*), and orangutans (*Pongo abelii*) used prior information about the length of two different pretzel sticks to select the longer one even when the pretzel sticks were covered partially such that the large stick appeared to be smaller than the short one before apes were allowed to choose (Karg et al. 2014).

In the most recent contribution, Krachun et al. (2016) presented chimpanzees with mirror images, which created the illusion of doubling the amount of available food. In particular, chimpanzees were presented with two open boxes in which different food quantities were placed. While the subjects were watching, a mirror was inserted in the box containing fewer food items, which doubled the perceived number of food items in this box. Three out of six chimpanzees passed this test by choosing the box without the mirror that appeared to contain fewer grapes but in reality contained more. One chimpanzee even passed a follow-up condition in which visual tracking was avoided by scrambling the two boxes out of sight. In this latter condition, subjects had to compare the perceived food quantities between the boxes after scrambling and select the apparently smaller quantity to obtain the larger one. The performance of this individual suggests that at least one chimpanzee can go beyond Piagetian conservation as she seemed to use the misleading appearance to identify a preferred reward instead of just ignoring the misleading appearance.

Krachun et al.’s (2016) study highlights the unique potential of mirrors for the investigation of AR discrimination. Standard plane mirrors reflect objects in the environment without significant distortions. It is, therefore, not surprising that mirror reflections can lead to misperceptions, for example, about the location of an object (Loveland 1986). Nevertheless, there is evidence that some primate species do not confuse the location of mirror reflections with the location of their physical referents. For example, various primate species are capable of using mirror images to locate otherwise nonvisible food items. Rhesus macaques (*Macaca mulatta*) learnt to use a mirror in a string-pulling paradigm to identify which of two strings was connected to a food reward (Brown et al. 1965). Even without explicit training, chimpanzees and Tonkean and long-tailed macaques (*M. tonkeana* and *M. fascicularis*) used mirror images to guide their hands toward a hidden food reward or colour mark (e.g., Anderson 1986; Menzel et al. 1985). In these studies, a large number of different target location was used. Chimpanzees also used a live video stream to guide their hands to the colour mark, even when the video stream was laterally reversed and inverted, and they discriminated between two screens showing either the live stream or the recorded images from previous sessions. When the screen was switched off at the beginning of control trials, the chimpanzees stopped reaching for the target immediately and only continued their search when the screen was switched on again (Menzel et al. 1985).

Self-recognition studies provide additional evidence that some primate species, in particular great apes, do not confuse mirror images with their referents. These studies show that apes show mirror-induced self-exploration without any explicit training (e.g., Gallup 1970; Köhler 1921; see also Anderson and Gallup 2015 for a recent review). A prominent experimental paradigm in this context is the so-called mark test. Here, a colour mark is placed on a usually unseen body part of the subject. Importantly, the subjects are unaware of this mark. Subsequently, subjects are presented with their mirror image. They pass the mirror test if they explore the mark or try to remove it. Chimpanzees (*P. troglodytes*), orangutans (*Pongo pygmaeus*), and gorillas (*G. gorilla*) have spontaneously passed the mark test (Anderson and Gallup 2015). These findings, together with those on mirror-guided search, suggest that some primate species, in particular apes, do not confuse mirror and video images with their referents at least when they receive continuous visuomotor feedback.

Shadows are another optical phenomenon that can provide an interesting contrast when compared to mirror reflections. Shadows, like mirror reflections, are causally related to a physical referent, that is, to the object that is casting the shadow. Moreover, shadows also resemble their referents though to a lesser extent than mirror reflections. In other words, mirror reflections and shadows both convey (causal) information about an object (e.g., the object’s outline) but the risk of confusion with the object differs considerably between the two. If an individual uses both of these optical cues similarly to locate the referent, one might argue that it is not the perceptual resemblance with the referent that is key, but the causal relation between the cue and its referent.

Despite their frequent occurrence, there is very little research to date investigating the kind of information that primates are capable of extracting from shadows (e.g., about the location of objects). Presented with their own silhouette cast on a wall, Guinea baboons (*Papio papio*) reacted aggressively in a few instances and there was no sign that they recognized their own shadow (Petit and Thierry 1994). Chimpanzees, in contrast, may relate their shadow to themselves, though the evidence to date is rather anecdotal. Boysen et al. (1994) presented two juvenile, enculturated chimpanzees with different situations in which they could locate objects by means of their shadows and recognize their own shadow. For instance, chimpanzees watched shadow plays showing either how a ball was passed behind the back of the chimpanzees from one experimenter to the next or how a hat was raised behind the chimpanzees creating the impression that the shadow of the chimpanzee wore a hat. In the latter case, especially one of the two chimpanzees sometimes produced self-directed responses when she could see the hat on top of her silhouette (e.g., moving her head presumably to detach her own silhouette from the shadow of the hat). In the case of the object shadow-recognition task, none of the chimpanzees preferentially oriented toward the experimenter who held the ball after the transfer. In general, a base rate of the measured behaviours was missing and no statistical inference could be drawn from these results. Therefore, it remains inconclusive whether chimpanzees can infer the relation between shadows and their own body or other objects in their environment.

In the current study, we examined what kind of information apes extracted from mirror images and shadows to locate hidden food items in space. First, we studied whether apes could estimate how far away an object was from their position based on a mirror image. Second, we tested whether apes were sensitive to the geometrical relation between the orientation of a mirror and the location of a reflected object. Third, we investigated what kind of cues apes used to recognize whether they looked at a mirror (as compared to a static picture of the mirror image). Finally, we examined whether apes would take advantage of shadows to locate a hidden food reward paying particular attention to the different risk of confusion with the referent’s location between shadows and mirror images. The null hypothesis throughout the paper is that apes’ performance is governed by an associative, reinforcement-based learning mechanism. According to this associative account, the close spatial contingency between visual cues (i.e., the mirror image or projected shadow) and the concealed food reward is sufficient for individuals to learn over multiple trials to use this cue to locate the reward. Importantly, even rapid learning of new spatial associations would be limited to the perceptual qualities of the presented cues and not involve any causal understanding of the optical relations involved (e.g., regarding the geometrical relation between the location of the mirror image and its referent).

## Experiment 1

In the first experiment, we investigated whether different ape species (chimpanzees, bonobos, and orangutans) would use mirror images and shadows to locate hidden food. We compared these cues to a baseline condition in which apes could see where the food was hidden and a control condition in which the apes did not receive any cue about the food location. We hypothesized that apes would perform significantly better in the mirror and shadow condition compared to the control condition if they were sensitive to the relation between these optical effects and their physical referents (i.e., the desired food reward). Moreover, we expected a similar performance between the mirror and shadow conditions and the visible displacement baseline condition.

### Materials and methods

#### Subjects

Eleven chimpanzees (*P. troglodytes*), six orangutans (*P. abelii*), and eight bonobos (*P. paniscus*) participated in this experiment. The subjects were housed at the Wolfgang Köhler Research Centre, Leipzig Zoo (Leipzig, Germany). The subjects were not deprived of food, and water was available ad libitum during testing. One of the 11 chimpanzees (Jeudi) stopped approaching the test site after two sessions and was, therefore, excluded from data analysis. Another chimpanzee (Frodo) only completed the mirror condition but was not available for the shadow condition. Our final sample consisted of 16 females and 8 males aged between 5 and 41 years (*M*_Age_ 20.3 years). Subjects had participated in various cognitive tasks prior to the study, none of which involving shadows or mirrors as source of information. All chimpanzees and orangutans (but not the bonobos) had previous experience with shadows in a pilot experiment in which they failed to use the shadows as a cue (except for two chimpanzees, Sandra and Jeudi, who scored significantly above chance at the individual level). In the pilot experiment, the hidden food reward was located on a transparent Plexiglas table and its shadow was projected to a surface underneath this table. In the current setup, the shadow of the reward was projected to a screen behind the hidden food reward in the apes’ line of sight. The individual performance in the shadow condition of Experiment 1 and 2 was not correlated with the performance in the pilot experiment (Spearman’s correlation: *r*_s_ = 0.240, *N* = 23, *p* = 0.267). Additionally, the bonobos who did not participate in the pilot experiment performed at similar levels compared to the other apes. Consequently, carry-over effects from the pilot experiment appear unlikely.

#### Apparatus

**Fig. 1:**
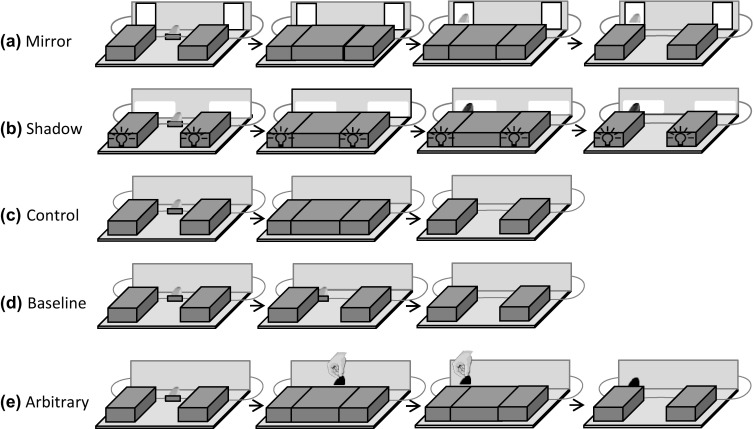
Experiment 1 and 2: illustration of the different conditions from the subjects’ point of view. Experiment 1 includes conditions (**a**–**d**); Experiment 2 includes conditions (**b**–**e**). A piece of banana (yellow oval) is placed on a displacement device. In all conditions except for the baseline condition, the food reward is then concealed. A silhouette (shadow condition), mirror image (mirror condition), or a black rubber patch (arbitrary condition) indicates the location of the concealed food reward. The rubber patch is displaced by the experimenter (tan shape = experimenter’s hand). (Color figure online)

The experimenter (*E*) sat behind the sliding platform (78.5 × 34 cm) facing the subject who was behind a transparent acrylic glass panel. This panel contained two or three horizontally aligned, circular holes (6 cm). The sliding platform was mounted 10.5 cm below the Plexiglas panel to ensure that the subjects could see all relevant parts of the platform, particularly the mirrors and projection areas for the shadows. On top of the sliding platform, there was a movable displacement device, a piece of PVC plastic material (7 × 3 cm), that served to displace the food placed on top of it (see Fig. 1). *E* could move the displacement device to the left or to the right side of the platform via a thin, transparent string (diameter 1 mm) attached to it. The two ends of the string were connected underneath the platform. *E* displaced the food by pulling the string underneath the platform, occluded from the subjects’ view. Thereby, the experimenter remained in a central position without leaning to the left or to the right. At the experimenter side of the platform, there was a screen mounted vertically to the platform (78 × 17 cm) onto which the shadows were cast (shadow condition) or the mirrors (15 × 15 cm) were attached (mirror condition). Three covers occluded the location of the food at the left, central, and right side of the platform (height × depth × width: 6.5 cm × 24.5 cm × 22/26 cm). In the shadow condition, two battery-powered bicycle lights (single LED, 0.5 W, 12 lx) were located under the left and right cover facing toward the screen onto which the shadows were cast. When the covers were in place, the subjects could still see the screen with the silhouette/mirror image in the back of the platform but not the food or the displacement device.

#### Design

We administered four different conditions: mirror, shadow, control, and baseline. In the mirror and shadows conditions, apes could use the mirror image and the shadow of the food, respectively, to locate the food (see Fig. 1a, b and Online Resource 2). In the control condition, apes did not receive any information about the location of the food (controlling for inadvertent cues that the subjects might pick up; see Fig. 1c) and in the baseline condition apes could directly observe how the food was moved under one of the two lateral covers (visible displacement baseline; see Fig. 1d).

We divided our sample into two groups according to the order in which they received the mirror and shadow condition. The mirror-first group received the mirror condition in the first four sessions and the shadow condition in the following four sessions, for the shadow-first group the order of conditions was reversed. Assignment to the groups was random except that we balanced the groups for age, sex, and species as much as possible (mirror-first group: *M*_Age_: 22.6 years, 7 females, 4 males; shadow-first group: *M*_Age_: 17.3 years, 9 females, 3 males). Three orangutans who were in the mirror-first group received a different experimental setup in the mirror condition (the sliding platform was mounted at a greater height from the floor). Due to this change in the setup, apes could barely see the food in the mirror when sitting in front of the Plexiglas panel. We repeated the mirror condition for these individuals after the shadow condition. Due to the deviation in procedure, we discarded the data of their first four sessions in the mirror condition and re-assigned them to the shadow-first group.

We administered 12 trials per session, each including 6 trials of the experimental condition (mirror or shadow), and 3 trials of the control and baseline condition. The reward was moved to the left in half of the trials and to the right in the other half. We pseudo-randomized the order of trials within a session with the restriction that the food was moved to the same side in no more than three consecutive trials. Moreover, subjects did not receive the same condition in more than four consecutive trials. All subjects completed 8 sessions, for a total of 24 trials per condition.

#### Procedure

We tested subjects individually. At the beginning of each trial, *E* moved the displacement device in the centre of the platform. In the mirror condition, *E* then mounted two mirrors (via magnets) at the screen on the left and right side of the platform facing toward the subject. In the shadow condition, *E* placed two lights on the platform in front of the left and right hole of the Plexiglas panel facing towards the vertical partition in the back. In the baseline and control condition, there were neither lights nor mirrors. Then *E* put the left and right covers on the platform (covering the two lamps in the shadow condition) and positioned a piece of banana on top of the displacement device in the centre. *E* covered the reward (except for the baseline condition) by putting the central cover on the platform and pulled the reward either to the left or to the right side via a string underneath the platform. *E* then lifted the central cover revealing that the food reward was gone. *E* pushed the platform forward allowing subjects to make a choice by inserting their hands into one of the two outer holes in the panel (for a picture of the setup, see Online Resource 1). *E* looked straight to the ground to avoid inadvertent cueing, while displacing the food until the subjects made their choice. Once the subjects had made their choice, *E* lifted the selected cover. If the choice was correct, the subjects received the piece of banana underneath the chosen cover. If the choice was incorrect (i.e., no food under the cover), *E* showed the apes where the food was hidden and discarded the food in the food bucket underneath the platform. In a few trials, apes’ selection was equivocal because they inserted their hands in both holes or they switched rapidly between the two options. In those cases, *E* pushed the platform back and forth again until the subject made an unambiguous decision.

#### Scoring and analysis

We scored the first choice of the subjects after *E* had pushed the sliding table to the subject. A second coder naïve to the hypotheses and theoretical background of the study scored 20% of all trials to assess inter-observer reliability, which was excellent (*Κ* = 0.99, *N* = 456, *p* < 0.001).

We used a generalized linear mixed model (GLMM; Baayen 2008) with binomial error structure and logit link function to analyse the effects of condition, sex, age, species, order of experimental conditions, and session on apes’ choices (correct/incorrect). We included these factors as fixed effects and subject ID as random effect. Additionally, to keep type I error rate at the nominal level of 5% (Barr et al. 2013; Schielzeth and Forstmeier 2009), we included all random slope components of condition (dummy coded), order of condition, and session. As an overall test of the effect of the predictor variables we compared the full model with a null model lacking the fixed effects but comprising of the same random effects structure as the full model (Forstmeier and Schielzeth 2011) using a likelihood ratio test (Dobson 2002). *p* values for the individual effects were based on likelihood ratio tests comparing the full with the respective reduced models (Barr et al. 2013; R function drop1).

We assessed model stability by comparing the estimates derived by a model based on all data with those estimates obtained from models with individual subjects excluded one at a time. This revealed the model to be stable with regard to the fixed effects. Overdispersion appeared to be no issue (dispersion parameter: 0.86).

Additionally, we used Wilcoxon signed-ranks tests to examine whether performance deviated significantly from the hypothetical chance level (*p* = 0.5). At the individual and for first trial analysis, we conducted binomial tests with a hypothetical probability of *p* = 0.5. All *p* values reported throughout this study are exact and two tailed.

### Results

We found evidence that apes used the mirror and shadow cues spontaneously to locate the food. Apes performed significantly better in the shadow and mirror condition compared to the control condition. Additionally, their performance in the shadow and mirror conditions was above chance levels. Analyses of the individual performances confirmed this result.

**Fig. 2:**
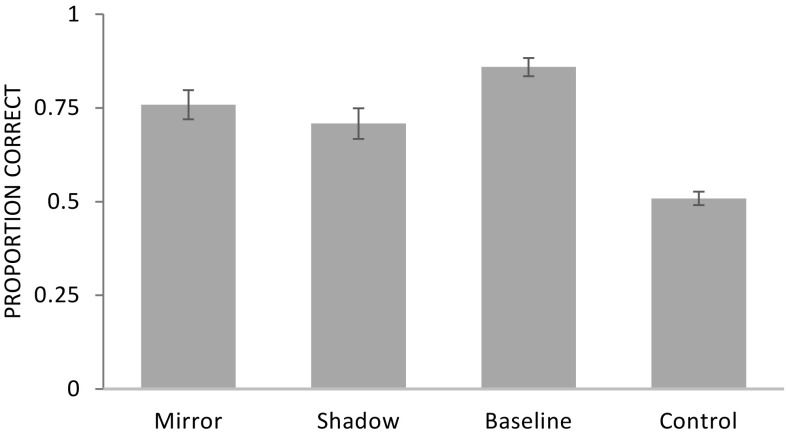
Experiment 1: proportion of correct trials (mean ± SE) as a function of condition

A GLMM comprising of the factors condition, order of mirror and shadow conditions, session, species, age, and sex was significant compared to a null model lacking these factors (likelihood ratio test: *χ*^2^ = 73.6, *df* = 9, *p* < 0.001; see Online Resource 1, for the model output). Condition had a significant effect on performance (*χ*^2^ = 67.7, *df* = 3, *p* < 0.001; see Fig. 2). Apes performed significantly better in the baseline (*χ*^2^ = 38.7, *df* = 1, *p* < 0.001), mirror (*χ*^2^ = 18.0, *df* = 1, *p* < 0.001), and shadow condition (1.08 ± 0.23, *χ*^2^ = 17.1, *df* = 1, *p* < 0.001) compared to the control condition. Moreover, apes performed better in the baseline condition compared to the shadow condition (*χ*^2^ = 8.1, *df* = 1, *p* = 0.004) but not compared to the mirror condition (*χ*^2^ = 1.8, *df* = 1, *p* = 0.180). There was no significant difference between shadow and mirror condition (*χ*^2^ = 2.95, *df* = 1, *p* = 0.086). We found no significant effect of species (*χ*^2^ = 2.3, *df* = 2, *p* = 0.322), the order of conditions, (*χ*^2^ = 0.1, *df* = 1, *p* = 0.816), session (*χ*^2^ = 1.0, *df* = 1, *p* = 0.328), age (*χ*^2^ = 0.002, *df* = 1, *p* = 0.964), or sex (*χ*^2^ = 1.7, *df* = 1, *p* = 0.195).

Apes performed significantly better than expected by chance in the baseline (mean ± SE: 0.86 ± 0.02; Wilcoxon signed-ranks test: *T*^+^ = 300, *N* = 24, *p* < 0.001), shadow (0.71 ± 0.04; *T*^+^ = 218, *N* = 21, *p* < 0.001), and mirror condition (0.76 ± 0.04; *T*^+^ = 209, *N* = 20, *p* < 0.001) but not in the control condition (0.51 ± 0.02; *T*^+^ = 129, *N* = 20, *p* = 0.380). This pattern of findings was already present in the first session of each experimental condition (shadow: *T*^+^ = 141, *N* = 18, *p* = 0.010; mirror: *T*^+^ = 132, *N* = 16, *p* < 0.001, mirror analysis without the data of the three orangutans that received another experimental setup initially). First trial analysis showed that apes performed above chance in the mirror condition (16 of 21 individuals chose correctly, binomial test: *p* = 0.027) but not in the shadow condition (15 of 23 individuals chose correctly, *p* = 0.210).

At the individual level, five out of eight bonobos, eight out of ten chimpanzees, and all six orangutans performed above chance in the baseline condition (binomial test, *p* < 0.05). In the shadow condition, four out of eight bonobos, four out of nine chimpanzees, and three out of six orangutans performed significantly above chance. In the mirror condition, three out of eight bonobos, six out of ten chimpanzees, and five out of six orangutans (for three of these orangutans the mirror condition was repeated) performed significantly above chance. In the control condition, none of the apes performed significantly above chance.

### Discussion

We found that some individuals of all examined ape species used shadows and mirror images of hidden food items as a cue to locate them. Other individuals did not use these cues. The reasons for the observed individual differences are unclear but attention to the problem situation, food motivation, and more specific cognitive differences might play a role here. We found no evidence that apes’ performance improved across sessions. On the contrary, we found that they used both cues already in their first session (i.e., within the first six trials with these cues), and mirror images already in their first trial.

However, these findings leave a number of open questions regarding apes’ understanding of these optical effects. With regard to the shadows, did they associate the silhouette with the location of the food based on rapid reinforcement learning? Or did the apes make use of the silhouette because they inferred location of the food reward as the physical referent of the shadow? If reinforcement learning was sufficient to explain apes’ performance, we expected that they would learn to use a perceptually similar and equally deterministic cue to locate the food within the same number of trials (see Experiment 2). With regard to the mirror images, first trial performance showed that reinforcement learning was not a viable explanation. However, in the latter case, apes might just have confused the mirror reflection with its physical referent. It is possible that they pointed toward the food they saw (in the mirror) without taking into account that it was just a reflection of the food reward. Thus, the question is whether apes discriminated between appearance and reality in the case of the mirror image.

Moreover, motion cues were available in both experimental conditions. The moving shadow or mirror reflection of the food reward was visible before the apes were allowed to choose. These motion cues may have directed their attention to the correct side which could potentially explain apes’ performance without invoking any higher-level processing of these optical effects. Were such motion cues necessary and/or sufficient to allow apes to exploit these optical cues?

The remaining experiments addressed these questions one by one. In the next experiment, we introduced a control condition, which shared perceptual features (including motion cues) and the reinforcement regime with the shadow condition but lacked the causal relation between cue and food location.

## Experiment 2

In this experiment, we examined whether reinforcement learning was sufficient to account for apes’ performance in the shadow condition of Experiment 1. To control for this possibility, we presented a sample of naïve chimpanzees with a novel control condition in addition to the shadow condition. In this arbitrary control condition (Call 2006), apes could use a perceptually similar and equally 100% deterministic cue (a black rubber patch of similar shape as the silhouette of the food reward) to locate the food. If reinforcement learning explained apes’ performance in the shadow condition, we expected a similar performance in both conditions. However, if the causal relation between the silhouette and food reward was relevant for chimpanzees’ performance, we expected better performance in the shadow condition compared to the arbitrary condition. In the latter condition, the experimenter moved the rubber patch to one side of the platform in full view of the subject. If the apes were sensitive to the causal relations involved, the experimenter’s intervention should lead them to discount the rubber patch as a predictor of the food location.

### Materials and methods

#### Subjects

Eleven chimpanzees participated in this experiment. These chimpanzees were naïve with regard to the current shadow setup. They participated in the shadow condition of the pilot experiment. Neither of these apes scored above chance in this pilot experiment. One chimpanzee (Corrie) did not approach the platform within four sessions and was, therefore, excluded from the study.

#### Apparatus

We used the same setup as in Experiment 1.

#### Design

We administered four conditions: baseline, control, and shadow condition as well as a new condition, the arbitrary control. As in Experiment 1, baseline and control trials were intermixed with the experimental conditions. The experimental conditions were blocked with half of individuals starting with the shadow condition and the other half with the arbitrary control condition. The two groups were counterbalanced for age and sex as much as possible (arbitrary first: *M*_Age_: 25.9 years, 4 females, 1 male; shadow first: *M*_Age_: 25.2 years, 3 females, 3 males). All other aspects of the design including the trial and session numbers were identical to Experiment 1.

#### Procedure

The baseline and control condition were identical to Experiment 1. The shadow condition was also similar to Experiment 1 with the exception that *E* waited 8 s after the food was covered before he called the subjects by their names (to ensure that the apes would pay attention to the platform) and displaced the food reward by means of a string underneath the sliding platform. The 8 s interval was introduced to keep the timing of cue presentation equal between the shadow condition and the novel arbitrary condition. In the arbitrary control condition, there were no lights under the lateral covers, and therefore, no shadows. However, *E* fixed a black rubber patch of approximately the same shape and size as the silhouette of the food reward (8 cm × 5.5 cm) on the vertical partition onto which the shadows were cast in the shadow condition to indicate the location of the food (see Fig. 1e). As in the shadow condition, *E* first placed the lateral covers on the platform, placed a piece of banana in the centre on top of the displacement device, and covered the reward by means of the central cover. In contrast to the shadow condition, *E* displaced the out-of-sight reward right away. Eight seconds after he had covered the food reward, *E* lifted the rubber patch from behind the platform while calling the subject. *E* then placed the rubber patch centrally on the subject’s side of the vertical partition, moved it to the side where the food was hidden, and fixated it to the vertical partition (with a magnet attached to the backside of the rubber patch). *E* then pushed the platform forward and the subject could make a choice. In all conditions, *E* avoided making eye contact with the subjects during the trials to reduce the probability that subjects would interpret these cues as communicative signals (Gómez 1996).

#### Scoring and analysis

Scoring was identical to Experiment 1. We used a GLMM with the same factors as in Experiment 1 (except for the factor species). The model was stable for all fixed effects when subjects were excluded one at a time. Overdispersion appeared to be no issue (dispersion parameter: 0.96).

### Results

We found that naïve chimpanzees used the shadows but not the rubber patch (arbitrary condition) as a cue to locate the food. In the shadow condition, apes performed better than in the control condition and above chance levels; neither of which applied for the arbitrary condition.

**Fig. 3:**
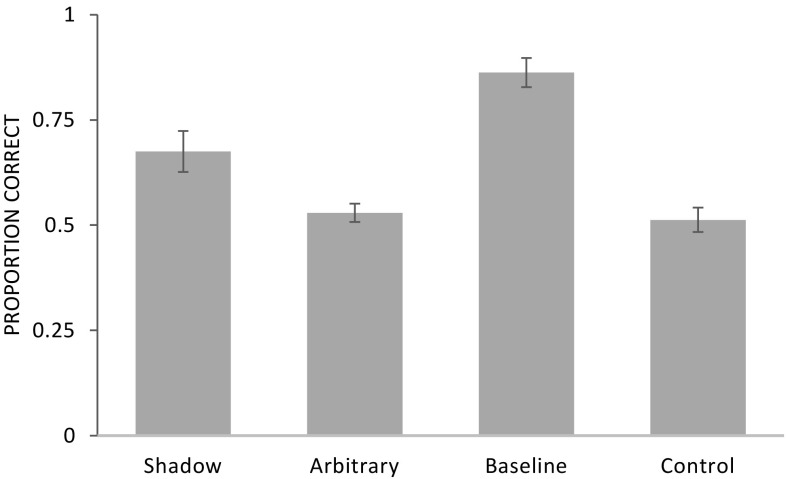
Experiment 2: proportion of correct trials (mean ± SE) as a function of condition

A GLMM comprising the factors condition, order of experimental conditions, session, age, and sex was significant compared to a null model lacking these factors (*χ*^2^ = 28.5, *df* = 7, *p* < 0.001; see Online Resource 1). Condition had a significant effect on performance (*χ*^2^ = 24.8, *df* = 3, *p* < 0.001; see Fig. 3). Apes performed significantly better in the baseline (*χ*^2^ = 19.2, *df* = 1, *p* < 0.001) and shadow condition (*χ*^2^ = 6.5, *df* = 1, *p* = 0.011) compared to the control condition. Likewise, apes performed better in the baseline condition (*χ*^2^ = 18.5, *df* = 1, *p* < 0.001) and the shadow condition (*χ*^2^ = 5.3, *df* = 1, *p* = 0.021) compared to the arbitrary condition. Apes performed also better in the baseline condition than the shadow condition (*χ*^2^ = 13.2, *df* = 1, *p* < 0.001). In contrast, there was no significant difference between the arbitrary and control condition (*χ*^2^ = 0.2, *df* = 1, *p* = 0.664). We found no significant effect of the order of experimental conditions (*χ*^2^ = 1.5, *df* = 1, *p* = 0.221), session (*χ*^2^ = 0.7, *df* = 1, *p* = 0.387), age (*χ*^2^ = 3.19, *df* = 1, *p* = 0.074), or sex (*χ*^2^ = 0.29, *df* = 1, *p* = 0.588).

Apes performed significantly better than the hypothetical chance level of 50% correct in the shadow (mean ± SE: 0.68 ± 0.05; *T*^+^ = 36, *N* = 8, *p* = 0.008) and baseline condition (0.86 ± 0.03; *T*^+^ = 55, *N* = 10, *p* = 0.002) but not in the arbitrary (0.53 ± 0.02; *T*^+^ = 33.5, *N* = 9, *p* = 0.254) or control condition (0.51 ± 0.03; *T*^+^ = 8.5, *N* = 5, *p* = 0.938). At the individual level, 3 out of 10 individuals scored significantly above chance in the shadow condition (binomial test *p* < 0.05), in contrast to the arbitrary and control condition in which none of the individuals did. In the baseline condition, 8 out of 10 subjects performed better than expected by chance.

### Discussion

We replicated the results obtained in Experiment 1. Naïve chimpanzees used the shadows as cue to locate food but they did not learn to use a perceptually very similar cue to locate the food within the same number of trials despite both cues being equally and fully deterministic.

One might argue that apes failed to use the rubber patch as a cue because it was only shown and moved 8 s after the hiding of the food. This time lag might have disrupted their causal perception of the situation (Michotte 1963; for a similar argument regarding the balance paradigm, see Povinelli 2011). Even though causal perception typically requires perceptual contact between two colliding objects (which is not the case here or in the balance task; see Hanus and Call 2008), we explicitly controlled for the changes in the temporal structure. Therefore, we introduced the same time lag also in the shadow condition. The temporal structure was identical in the two conditions; nevertheless, apes treated them differently. Likewise, both conditions involved motion cues (i.e., the movement of the silhouette in the shadow condition and the experimenter moving the rubber patch in the arbitrary condition), which were, therefore, insufficient to explain apes’ performance. However, it is possible that the “self-propelledness” of the moving silhouette made this cue more salient to the apes.

In a recent review of the literature on inferential abilities of nonhuman animals (Völter and Call 2017), we came to the conclusion that temporal structure helps great apes to discount alternative causes. Temporal structure was available in the current task (the cue was moved before apes could see that the food was not in the central position anymore) but it could not account for the differences between conditions either. At the time of choice, apes could see a perceptually similar situation in the shadow and arbitrary condition: a dark cue of similar shape as the food reward was visible on one side of the platform. The crucial difference between the two conditions was what happened before these cues were presented: in the shadow condition, the shadow appeared without any obvious intervention of the experimenter. In the arbitrary condition, the experimenter moved the rubber patch to one side of the platform. It is precisely this intervention by the experimenter that might have altered the situation for the apes and that led them to discount the rubber patch as relevant piece of information regarding the location of the food.

In sum, the current experiment suggests that apes used the silhouette of a piece of food by inferring its physical referent. For the remainder of this paper, we focused on the information apes can extract from mirror images.

## Experiment 3

In this experiment, we examined whether apes (like adult humans, Higashiyama and Shimono 2004) were capable of assessing the distance of an object from their own position based on its mirror reflection. If they were able to extract depth information, we hypothesized, they would point toward the location of the (hidden) food reward rather than toward its mirror reflection. Conversely, if they confused the mirror reflection with its physical referent (i.e., the hidden food), we expected that the apes would point toward the mirror image regardless of the food location.

### Materials and methods

#### Subjects

All apes who scored significantly above chance in the mirror condition of Experiment 1 participated in the pretest of the current experiment (*N* = 14, three bonobos, six chimpanzees, and five orangutans).

#### Apparatus

**Fig. 4:**
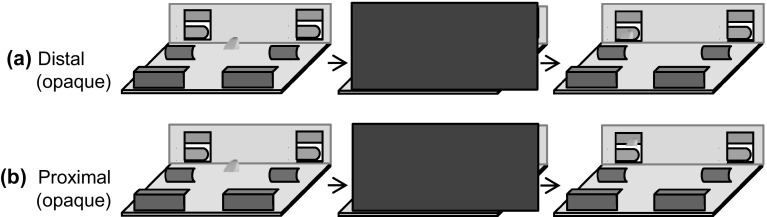
Experiment 3: illustration of the setup in the opaque condition from the subjects’ perspective. The food was hidden either behind one of the (green) distal (**a**) or (brown) proximal screens (**b**). Two mirrors in the back of the platform indicate the final position of the food reward (yellow oval). (Color figure online)

*E* sat behind the sliding platform facing the apes, who were located behind a metal mesh panel. As in Experiment 1, mirrors were mounted on the vertical partition located in the back of the platform. We used four screens as potential hiding places of the food reward (a piece of banana). Two of these screens were close to the mesh panel (proximal screens) and two were further away from it (distal screens). The screens were located on the left and right side of the platform (see Fig. 4 and see Online Resource 1). The food could thus be hidden on the left or right side and proximal or distant from the apes’ location behind the mesh panel. The screens were of different shape and colour. The proximal screens had the shape of an inverted *L* (19 cm × 3.5 cm × 5 cm); the distal screens were C-shaped (15 cm × 6 cm). In the pretest and the transparent condition of the test phase, all screens were transparent; in the opaque condition, the proximal screens were brown and the distal screens were green. For this experiment, we tilted the sliding platform so that the side closest to the apes was lower in height than to the distal side. Due to the inclination of the platform, the proximal screens were beneath the mesh panel when *E* pushed the platform forward. We used an inclined platform to reduce the likelihood that the apes would touch the proximal screen accidentally while pointing toward the distal screen.

#### Design

In the pretest, there were no mirrors and the screens were transparent. The pretest served to ensure that apes would try to access food within-reach by directly removing or touching the proximal screen or pointing downwards toward the proximal screen while they would point straight ahead toward the out-of-reach food without touching the unbaited, proximal screen. Subjects received 12 trials per session, 6 trials with the food in the proximal and distal location. Apes received a maximum of three sessions. If they did not meet the criterion (details below) within these three sessions, they were excluded from this experiment. Two (out of three) bonobos, all six chimpanzees, and four (out of five) orangutans (*N* = 12) met the criterion.

The subjects who passed the pretest entered the test phase. In the test, two mirrors were mounted on the left and right side of the platform. We used a 2 × 2 within-subject design: *E* hid the reward either in a proximal or distal location from the apes and either behind transparent or opaque screens. We administered three trials per cell (proximal/distal × transparent/opaque) and session for a total of 12 trials per session. The side of the food reward was counterbalanced across trials and the order was randomized with the restriction that food was hidden at the same side for a maximum of three consecutive trials. Subjects received four sessions for a total of 12 trials per cell.

#### Procedure

At the start of each pretest and test trial, *E* positioned the transparent or opaque (in test trials only) screens on the platform, occluded the platform and hid the food reward behind one of the four screens. *E*’s hand visited every screen while hiding the food in the following order: distal left, proximal left, proximal right, and distal right. After the hiding of the bait, *E* removed the occluder from the platform and pushed the platform forward. Apes could make a choice by sticking their fingers through the mesh panel (see Online Resource 3). We administered differential reinforcement for their lateral choice (correct or incorrect side) but not for the distance of the food reward. If the apes touched or pointed downwards to the proximal screen, *E* first lifted the indicated screen and passed food located behind it to the apes. If the apes did not touch the proximal screen and pointed toward the distal screen, *E* lifted the distal screen first. If the food was not located behind the indicated screen, *E* lifted first the indicated screen, then the other screen on the same side, and passed the food behind the latter screen to the subjects.

#### Scoring and analysis

We scored whether the apes pointed to the correct side and whether they touched/pointed toward the proximal or the distal screen. The form of the pointing gesture varied between individuals with some apes pointing with only one finger and others using their whole hand. Irrespective of these differences, we coded the direction of the extended finger(s). We scored trials as correct when the apes touched or pointed toward the (baited) proximal screen in the proximal condition and when they pointed toward the (baited) distal screen in the distal condition. The criterion for the pretest was to score five out of six trials correct in both conditions within a session. A second coder naïve to the hypotheses and theoretical background scored 21% of all trials from the recorded video material to establish inter-observer reliability. The videotapes showed the actions of the experimenter, the baiting status of the platform, and the choices of the subjects. Inter-observer reliability was excellent according to the guidelines by Fleiss (1981; correct side: *Κ* = 0.95, *N* = 120, *p* < 0.001; touching/pointing: *Κ* = 0.84, *N* = 120, *p* < 0.001).

We used a GLMM (Baayen 2008) with binomial error structure and logit link function to analyse the effects of visibility, distance of the bait, species, trial number, and session on apes’ lateral choices (correct/incorrect) and pointing (proximal/distal). Regarding model stability, the models were stable for all fixed effect except for visibility. We found considerable variation for visibility in the lateral choice analysis (estimates of visibility when subjects were excluded one at a time: orig. − 3.9, min. − 20.6, max. − 3.7). The variation of estimates indicates that the effect of visibility got stronger if certain individuals were excluded. Overdispersion appeared to be no issue (dispersion parameter: correct side: 0.79, distance: 0.82).

### Results

We found evidence that apes extracted depth information from the mirror images. More specifically, apes adjusted their response to the distance of the food by pointing toward the proximal or the distal screen depending on the location of the food and even when the food was only visible in the mirror.

We first analysed whether apes chose the correct side across conditions. A GLMM with the factors visibility, distance, trial number, session, and species was significant compared to a null model without these fixed effects (*χ*^2^ = 38.5, *df* = 6, *p* < 0.001; see Online Resource 1). Apes performed significantly better when the food was directly visible than when it was only visible via the mirror (*χ*^2^ = 24.1, *df* = 1, *p* < 0.001). Moreover, subjects performed better when the food was distal compared to when it was proximal (*χ*^2^ = 16.3, *df* = 1, *p* < 0.001). In contrast, we found no effects of trial number (*χ*^2^ = 0.1, *df* = 1, *p* = 0.744), session (*χ*^2^ = 0.2, *df* = 1, *p* = 0.630), or species (*χ*^2^ = 0.3, *df* = 2, *p* = 0.870) on performance.

In all conditions, apes performed significantly better (by selecting the correct side) than expected by chance (clear-proximal: mean ± SE: 0.99 ± 0.01; *T*^+^ = 78, *N* = 12, *p* < 0.001; clear distal: 1.00 ± 0.00; *T*^+^ = 78, *N* = 12, *p* < 0.001; opaque proximal: 0.77 ± 0.05; *T*^+^ = 75.5, *N* = 12, *p* = 0.002; opaque distal: 0.94 ± 0.02; *T*^+^ = 78, *N* = 12, *p* < 0.001). At the individual level, 12 out of 13 individuals performed significantly above chance in the opaque condition and all 13 individuals performed above chance in the clear condition (binomial test: *p* < 0.05).

**Fig. 5:**
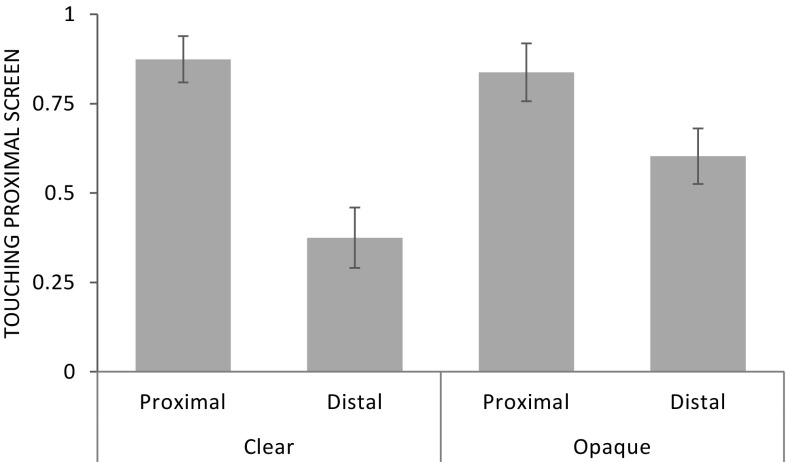
Experiment 3: proportion of trials (mean ± SE) in which apes touched or pointed toward the proximal screen as a function of reward position (proximal/distal) and reward visibility (clear/opaque screens)

Next, we analysed whether apes adjusted their response to the distance of the food by touching or pointing toward the proximal screen or pointing toward the distal screen. For this analysis, we only used trials in which apes chose the correct side because there was no reason to expect that apes would differentiate between the proximal and distal condition when they pointed to the incorrect side. A GLMM including visibility, distance, and the two-way interaction between these factors as well as session and species was significant compared to a null model (*χ*^2^ = 34.6, *df* = 6, *p* < 0.001; see Online Resource 1). We found a significant interaction between visibility of the food and its distance (*χ*^2^ = 5.6, *df* = 1, *p* = 0.017; see Fig. 5) but no significant effects of session (*χ*^2^ = 1.34, *df* = 1, *p* = 0.248) or species (*χ*^2^ = 0.9, *df* = 2, *p* = 0.633). Post hoc tests show that apes were more likely to point toward the proximal screen when the food was behind it than when it was located behind the distal screen both in the clear condition (*χ*^2^ = 19.2, *df* = 1, *p* < 0.001) and in the opaque condition (*χ*^2^ = 15.7, *df* = 1, *p* < 0.001) even though the effect in the latter condition was smaller. This difference between the proximal (clear proximal: mean ± SE: 0.86 ± 0.09; opaque proximal: 0.79 ± 0.12) and distal condition (clear distal: 0.27 ± 0.10; opaque distal: 0.48 ± 0.13) was already present in the first test session (clear: *T*^+^ = 36, *N* = 8, *p* = 0.008; opaque: *T*^+^ = 28, *N* = 7, *p* = 0.016). The performance was similar when all trials were considered including trials in which apes chose the incorrect side (mean ± SE: clear-proximal: 0.88 ± 0.06; opaque proximal: 0.81 ± 0.08; clear distal: 0.38 ± 0.08; opaque distal: 0.63 ± 0.08).

### Discussion

First, the current results replicate and extend the findings of Experiment 1. Eleven out of 12 apes chose the correct side above chance levels in the opaque condition indicating that they paid attention to the mirror images. Apes used the mirror to locate the food even in the absence of motion cues that were available in Experiment 1. Besides, apes performed better when the food was in the distal position than in the proximal position. These findings can be attributed to the fact that the mirror image of the food in the distal position was larger (the food was closer to mirror), and therefore, more salient.

Second and more importantly, apes further modulated their response according to the details of the mirror image suggesting that they did not confuse the mirror image with its physical referent. Depending on the distance of the food relative to them, apes tried to access the food directly by touching, removing, or pointing toward the proximal screen or they pointed toward the distal screen. Crucially, apes could extract such depth information about the distance of the reward from the mirror image alone. Apes might have used different pieces of information to extract the depth information from the mirror. For instance, they might have used the vertical position of the mirror image of the food reward (the lower the mirror image the closer the food was to the mirror), the proximity of the food reward to one of the opaque screens (i.e., matching the colour and shape of the screen next to the food in the mirror to the screens on the platform), or the size of the mirror image of the food (the larger the mirror image the closer the food was to the mirror). These possibilities are of course not mutually exclusive and subjects may have exploited a combination of these different sources of information. Importantly, however, reinforcement learning was not sufficient to explain apes’ pointing response because we did not apply differential reinforcement for apes’ pointing style to discriminate between proximal and distal screens.

## Experiment 4

In this experiment, we examined whether apes could use another aspect of the geometrical relations between mirror reflections and their physical referents, the law of reflection, which states that the angle of incident equals the angle of reflection. We presented apes with a situation, in which the orientation of the mirror predicted the location of the food reward and examined whether the apes would use this information when searching for the food. Naive human observers are sensitive to the law of reflection even though their predictions are not perfect (e.g., Bertamini et al. 2003; Bertamini and Wynne 2010; Croucher et al. 2002; Hecht et al. 2005). We compared apes’ performance in the mirror condition to a control condition in which the orientation of a static picture of the food reward predicted the location of the food reward. The mirror images differed in several ways from the picture stimuli that just showed the food reward on white background, including the complexity of the depicted scene in the mirror and the dynamic nature of the mirror image that became apparent when subjects moved their heads or the platform was moved. We hypothesized that if the apes took the geometrical relation between mirror and referent into consideration, they would adjust their choices to the orientation of the mirror but not to the orientation of the picture.

### Materials and methods

#### Subjects

All apes who scored significantly above chance in the mirror condition of Experiment 1 participated (*N* = 14, three bonobos, six chimpanzees, and five orangutans). One orangutan (Dokana) stopped participating after five sessions. For this reason, we only analysed the data of her first experimental condition (picture condition) tested against the hypothetical chance level.

#### Apparatus

E sat behind the sliding platform facing the apes who were located behind a Plexiglas panel. The Plexiglas panel contained two big, horizontally aligned holes (diameter: 6 cm) serving as response locations. In the centre of the panel there was a small hole (diameter 1 cm) for a juice dispenser (an infusion bottle mounted above the panel with a hose leading to the hole in the panel). During trials, the apes received diluted grape juice (concentration 1:3) to keep their angle of vision constant and centred. The flow of juice was reduced or terminated between trials. As in the previous experiments, apes were required to locate a hidden piece of banana. We used mirrors and pictures of a banana slice as cues. The pictures of the banana slice were taken from a similar angle as they appeared in the mirror from the apes’ perspective. The picture of the banana slice (3.3 cm × 4.0 cm; similar in size to the mirror image of the banana slice in the congruent condition) was then isolated, printed on white background, and glued to the backside of one of the mirrors. The distractor cue in the picture condition consisted of a blank, white paper glued to the backside of the second mirror.

The two mirrors/pictures (7.5 cm × 15 cm) were mounted on rotatable stands close to the experimenter side of the platform (for pictures and videos of the setup, see Online Resource 1 and 4). The mirrors/pictures were 11.5 cm apart from each other. When the mirrors on the stands were turned the angle of incidence changed. The two end positions of each stand (and therefore also the angles of incidence) were pre-determined by two screws that protruded from the platform. The food could be hidden behind two screens (the L-shaped screens from Experiment 3) located in front of the left and right response holes in the Plexiglas panel.

In the congruent position, the mirrors/pictures were turned by 25° towards the outside. In this case, the subjects could see behind the screens on the same side as the respective mirror. That is, they could see the mirror image of the food on the same side of the platform where the food was actually located. The same reinforcement regime was applied to the picture condition: when the picture of the banana slice was oriented outwards, the food was located behind the screen on the same side.

In the incongruent position, the mirrors and pictures were turned by 40° towards the inside of the platform. In this case, the mirrors showed what was behind the screen on the opposite side. That is, the subjects could see in the left mirror when the food was behind the right screen and vice versa. Again, the same applied to the picture condition: when facing inwards, the picture of the food indicated that food was located on the opposite side.

#### Design

We used a 2 × 2 within-subject design: the apes either received mirror or pictures as cues and the orientation of these cues was either congruent (facing outwards, the mirror image/picture of the food indicated the presence of food on the same side) or incongruent (facing inwards, the mirror image/picture indicated the presence of food on the opposite side). We blocked the mirror and picture condition into 4 consecutive sessions of 12 trials each. The order of condition was counterbalanced across subjects while balancing the two groups as much as possible regarding species, age, and sex (mirror first: *M*_Age_: 18.4 years, 2 males, 6 females; image first: *M*_Age_: 23.8 years, 2 males, 4 females). Per session, subjects received three blocks of four trials each. Within a block, the side of the food and congruence of the cue was fully crossed and the order of trials within a block was randomized. Subjects received 8 sessions for a total of 24 trials per cell.

#### Procedure

At the start of each trial, the mirror/pictures were oriented parallel to the platform. Then, the subject’s view to the platform was occluded. *E* hid the reward behind one of the two screens by visiting first the left and then the right screen leaving the food behind one of the screens. *E* exchanged the pictures if necessary and turned the mirrors/pictures either outwards (congruent condition) or inwards (incongruent condition). Subsequently, he lifted the occluder and pushed the platform forward. The apes could now make a choice and *E* lifted the screen indicated by the subject. Subjects received the food reward if they chose correctly. If they decided for the incorrect side, *E* first lifted the unbaited screen, then the baited screen, and discarded the food.

#### Scoring and analysis

We scored whether or not subjects chose the side with the mirror image or picture of the food. Again, we used a GLMM (Baayen 2008) with binomial error structure and logit link function to analyse the effects of cue type, congruence, order of cue type presentation, all two-way and three-way interactions between these factors, and species on subjects’ choices. The models were stable for the effects of cue type, congruence, order of condition, and the interactions between these factors. Overdispersion was no issue (dispersion parameter: 0.97).

### Results

Apes selected the side where they could see the mirror image of the food more often when the mirrors were oriented outwards (i.e., directed toward the screens on the same side) than inwards (i.e., directed toward the screens on the opposite side). The orientation of the picture, in contrast, had no such effect on apes’ performance.

**Fig. 6:**
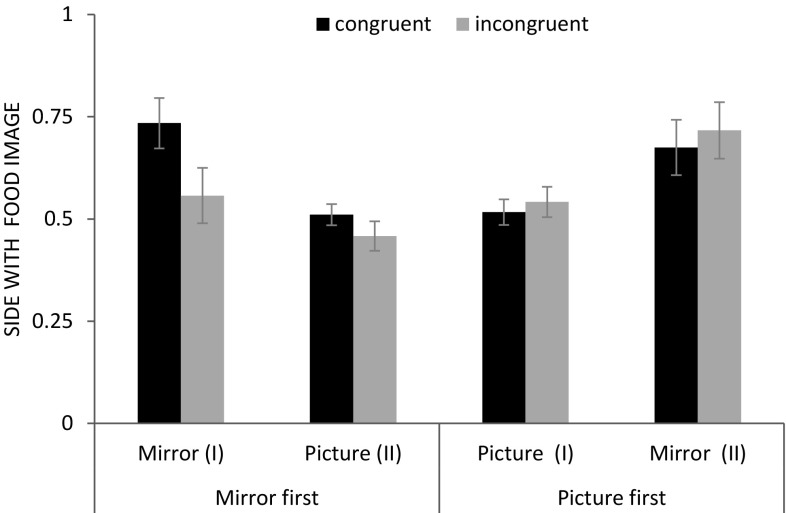
Experiment 4: proportion of trials (mean ± SE) in which subjects chose the side where they could see the food as a function of condition, cue type, and order of cue-type presentation. In the congruent condition, the food is located on the same side as the food image, whereas in the incongruent condition it is located on the opposite side

In a GLMM containing the factors cue type (mirror, picture), congruence (congruent, incongruent), order of cue-type condition, the two- and three-way interactions between these factors as well as species was significant compared to a null model lacking these factors (likelihood ratio test: *χ*^2^ = 27.9, *df* = 9, *p* = 0.001; see Online Resource 1). The three-way interaction between cue type, congruence, and the order of the cue-type presentation had a significant effect on apes’ performance (*χ*^2^ = 8.7, *df* = 1, *p* = 0.003; see Fig. 6) whereas species had not (*χ*^2^ = 2.5, *df* = 2, *p* = 0.287).

Focusing on the between-subject manipulation (first cue type presented to each subject), we found a significant two-way interaction between cue type and congruence (*χ*^2^ = 7.4, *df* = 1, *p* = 0.006). Subjects decided more often for the side where they could see the food when the mirrors were in congruent rather than incongruent orientation (*χ*^2^ = 9.4, *df* = 1, *p* = 0.002). In contrast, the congruence of the picture had no significant effect on performance (*χ*^2^ = 0.2, *df* = 1, *p* = 0.659).

Focusing on the within-subject manipulation, we found a significant interaction between cue type and congruence for the individuals who started with the mirror condition (*χ*^2^ = 4.8, *df* = 1, *p* = 0.029). These individuals showed an effect of congruence in the mirror condition (see above) but not in the picture condition (*χ*^2^ = 1.0, *df* = 1, *p* = 0.307). In contrast, individuals who started with the picture condition showed no significant interaction between cue type and congruence (*χ*^2^ = 0.1, *df* = 1, *p* = 0.776). The latter individuals looked more often to the side where they could see the food in the mirror condition (presented after the picture condition) compared to the initial picture condition (*χ*^2^ = 4.1, *df* = 1, *p* = 0.042) but their performance was not significantly affected by the orientation of these cues (*χ*^2^ = 0.6, *df* = 1, *p* = 0.438).

Looking at the first session (full–null model comparison: *χ*^2^ = 11.2, *df* = 5, *p* = 0.047; see Online Resource 1), we found no significant interaction between cue type and congruence (*χ*^2^ = 1.79, *df* = 1, *p* = 0.181). We then removed the interaction but we found no significant main effects of cue type (*χ*^2^ = 0.02, *df* = 1, *p* = 0.887) or congruence (*χ*^2^ = 0.5, *df* = 1, *p* = 0.472). Species, however, had a significant effect on performance (*χ*^2^ = 8.83, *df* = 2, *p* = 0.012) with chimpanzees deciding more often for the side were they could see the food than bonobos (*χ*^2^ = 8.8, *df* = 1, *p* = 0.003). There were no significant differences between orangutans and bonobos (*χ*^2^ = 2.0, *df* = 1, *p* = 0.162) or chimpanzees (*χ*^2^ = 2.5, *df* = 1, *p* = 0.117).

In line with these findings, apes who started with the mirror condition selected the side where they could see the mirror image of the food more often than expected by chance with a congruent mirror orientation (mean ± SE: 0.73 ± 0.06; *T*^+^ = 36, *N* = 8, *p* = 0.008) but not with an incongruent mirror orientation (0.56 ± 0.07; *T*^+^ = 21, *N* = 8, *p* = 0.742). In contrast, apes who started with the picture condition did not select the side with the picture of the food significantly more often in the picture congruent (0.52 ± 0.03; *T*^+^ = 6, *N* = 4, *p* = 1) or incongruent condition (0.54 ± 0.04; *T*^+^ = 12, *N* = 6, *p* = 0.906).

### Discussion

In this experiment, we examined whether apes were sensitive to the geometrical relation between the orientation of a mirror and mirrored objects. Even though apes initially (i.e., in session 1) showed no significant preference for the mirror image of a banana slice compared to the picture, apes learned to discriminate between the congruent and incongruent mirror orientation within four sessions. In contrast, apes that started with the picture condition did not learn to use the picture orientation as predictor of the food location.

However, irrespective of the order of conditions, apes’ performance in the incongruent condition did not deviate significantly from chance. This finding could be related to a limited understanding of the optical relations involved or it might hint to an inhibition problem. For example, the difference in performance between the congruent and incongruent condition might be attributed to larger size of the mirror reflections of the food reward in the congruent condition compared to the incongruent condition. Alternatively, apes’ prepotent response in this situation might have been to select the side where they could see the image of the reward. In the incongruent condition, however, pointing to the location of the hidden food reward required them to select the side where they could not see the image of the food reward.

Additionally, the within-subject comparison was confounded by an order effect. Individuals who started with the picture condition, unlike those who started with the mirror condition, did not learn to use the mirror orientation as discriminatory stimulus. Given this order effect, the within-subject comparison needs to be interpreted with caution. We can only speculate why apes who started with the picture condition did not learn to use the mirror orientation as discriminatory cue. The picture condition might have been confusing for the apes given that there was no causal connection between the orientation of the picture and the location of the food. This may have turned the task into a reverse contingency task, which is notoriously difficult for apes (e.g., Albiach-Serrano and Call 2014; Boysen and Berntson 1995; Vlamings et al. 2006). Frustration induced by such a counterintuitive task may have masked apes’ performance in the subsequent mirror condition.

Finally, one might object that the picture stimulus possibly was not as salient as the mirror image. If that was true, one might expect that apes were more drawn to the mirror image of the food right from the beginning of the experiment. However, we found no differences between the mirror and picture cue types in session 1. Nevertheless, we tried to improve the picture control stimulus in the final experiment by enhancing the similarity between the mirror image and a picture control stimulus.

## Experiment 5

In the final experiment, we addressed the question of whether prior experience with the properties of mirrors (as opposed to those of static pictures) affected chimpanzees’ interpretation and usage of these cues. We hypothesized that subjects might extract information from mirror images conditional on their experience with mirror properties. In this experiment, we focused on experience at two different timescales: the experience chimpanzees had acquired during Experiments 1, 3, and 4 and a direct demonstration of the dynamic properties of mirrors before each trial. We expected that mirror-experienced chimpanzees would use the mirror images more frequently to locate hidden food than mirror-naïve ones. Further we expected that apes would use a mirror image to locate its physical referent more effectively if they received a mirror demonstration before the onset of a trial. Conversely, we hypothesized that apes would not use this cue to the same extent if they received a demonstration showing that the cue was merely a static picture of the mirror image.

### Materials and methods

#### Subjects

All six chimpanzees who scored significantly above chance in the mirror condition of Experiment 1 and eight mirror-naïve chimpanzees participated in this experiment.

#### Apparatus

We used the same sliding platform and lateral covers as in the mirror condition of Experiment 1. We also used the Plexiglas panel with two big, lateral holes and a small, central hole for a juice dispenser of Experiment 4. Apes received juice during the trials to keep them centred and their angle of view stable.

The subjects were presented with two different cue types in the current experiment: mirrors and pictures depicting the mirror images when the mirrors were in their final position. We used two different sets of pictures depending on whether the food was hidden behind the left or right cover on the platform. In each set, one of the pictures showed a piece of banana on top of the displacement device and hidden behind one of the lateral covers, whereas the other picture showed the backside of the opposite cover without food. The photographs were taken from the same position and angle that the apes had while drinking juice from the dispenser, adjusted for luminosity to mimic the appearance of the mirror image, and printed on matt, photographic paper (see Online Resource 1, for an example of the experimental stimuli). These picture stimuli were glued to the backside of the mirrors (15 × 15 cm).

#### Design

We used a 2 × 2 within-subject design: the apes either received a mirror or picture demonstration before each trial and they were presented with mirror or pictures as cues within each. Apes received eight 12-trial sessions in total. Each session consisted of three blocks of four trials each. In each block, food location (left, right) and cue type (mirror, picture) were completely crossed. The order of trials within a block was randomized. The mirror/picture demonstration conditions were blocked into four consecutive sessions. Half of the subjects started with the mirror demonstration, while the other half started with the picture demonstration. The two groups were counterbalanced for mirror experience, age, and sex as much as possible (mirror first: *M*_Age_: 25.1 years, 5 females, 2 males; picture first: *M*_Age_: 25.3 years, 4 females, 3 males).

#### Procedure

**Fig. 7:**
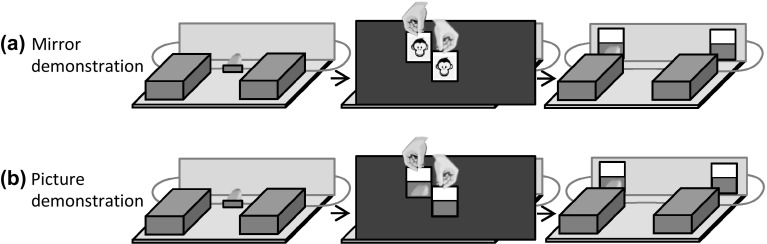
Experiment 5: illustration of the setup from the subjects’ perspective. Subjects were presented either with mirrors (**a**) or pictures of the final mirror images (**b**) while the platform was occluded. In the final stage of the procedure, subjects saw either two mirrors or pictures of the mirror images (independent of the cue type shown before)

At the start of each trial, *E* placed two lateral covers on the platform (i.e., the hiding places for the food rewards) and placed a piece of banana on the displacement device in the centre of the platform (see Fig. 7). *E* occluded the whole platform from the subjects’ view by placing a big screen on the platform and moved the baited displacement device via a string underneath the platform under one of the lateral covers. Then, *E* presented the apes either with two mirrors or with two pictures. *E* held the pictures or mirrors on top of each other directly in front of the subjects’ eyes for 3–5 s, while they were drinking juice. In the mirror demonstration (see Fig. 7a), apes could see themselves in the mirror when *E* held the mirrors in front of their face. In the picture demonstration (see Fig. 7b), apes saw pictures of the mirror images in their final position taken from the apes’ perspective. The experimenter then took these cues back, behind the screen on the platform and mounted them (with magnets) to the vertical partition in the back of the platform. Depending on the condition, *E* would either use the same cue that he had shown to the apes before (mirror or picture) or he flipped the cues over to invert the cue type (from mirror to picture or vice versa; see Online Resource 5). Irrespective of the cue type, the mirror image or the picture of the banana slice indicated the correct side. *E* pushed the platform forward and apes were allowed to make their choice by pointing through one of two lateral holes in the Plexiglas panel. After apes had made their choice, *E* removed the cues from the vertical partition (to avoid that subjects would receive additional feedback from the mirror images in between trials) and removed the lateral cover indicated by the apes. Apes received the food if they had chosen correctly; otherwise, *E* discarded the food.

#### Scoring and analysis

We scored whether subjects chose the side where they could see the food (in the mirror or on the picture) or not. Again, we used a GLMM (Baayen 2008) with binomial error structure and logit link function to analyse the effects of cue type, demonstration, mirror experience (six chimpanzees had used the mirror images before to locate food, eight chimpanzees were not experienced with mirrors), all two-way and three-way interactions between these factors, and order of demonstration on apes’ choices. The models were stable with regard to the fixed effects. Overdispersion appeared to be no issue (dispersion parameter: 0.92).

### Results

We found that mirror-experienced apes overall performed better with mirrors as cues than with pictures irrespective of the (mirror or picture) demonstration they received before each trial. However, in their first session, experienced apes performed better than naïve individuals after they had received a mirror demonstration but not after a picture demonstrations irrespective of the cue type.

A GLMM with the factors mirror experience, demonstration, cue type, and all interactions between these factors as well as the order of demonstration was significant compared to the null model lacking these factors (*χ*^2^ = 17.2, *df* = 8, *p* = 0.028). However, the three-way interaction between mirror experience, demonstration, and cue type was not significant (*χ*^2^ = 0.01, *df* = 1, *p* = 0.905).

**Fig. 8:**
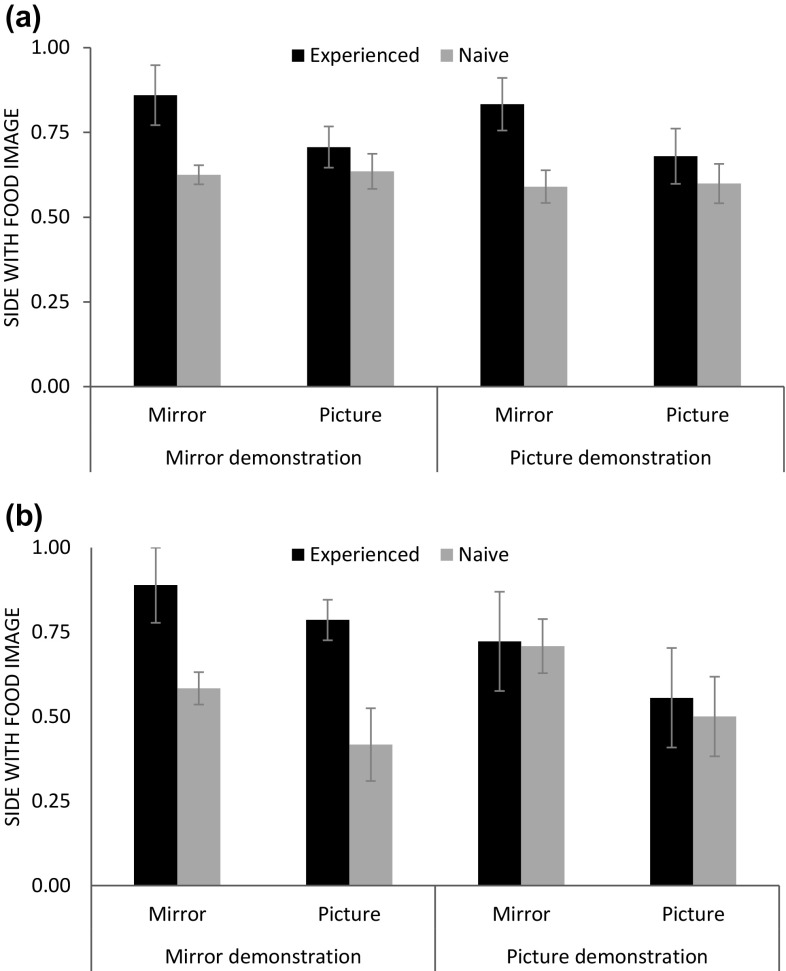
Experiment 5: proportion of trials (mean ± SE) in which subjects chose the side with the food image (mirror or picture) as function of mirror experience and demonstration. **a** Overall performance; **b** first test session

A reduced model without this three-way interaction was significant compared to a null model (*χ*^2^ = 17.2, *df* = 7, *p* = 0.016; see Table S7). The interaction between cue type and mirror experience was significant (*χ*^2^ = 7.4, *df* = 1, *p* = 0.006; see Fig. 8a). The mirror-experienced subjects performed better with mirrors compared to pictures of the mirror images (*χ*^2^ = 4.5, *df* = 1, *p* = 0.034) in contrast to the mirror-naïve individuals (*χ*^2^ = 0.04, *df* = 1, *p* = 0.846). There were no significant interactions between cue type and demonstration (*χ*^2^ = 0.003, *df* = 1, *p* = 0.956) or mirror experience and demonstration (*χ*^2^ = 0.0001, *df* = 1, *p* = 0.994). Neither were there significant main effects of demonstration (*χ*^2^ = 0.9, *df* = 1, *p* = 0.347) or order of demonstration (*χ*^2^ = 0.9, *df* = 1, *p* = 0.342).

Apes might have learned over sessions that the cue they looked at in the demonstration was different from the one they looked at in the end of a trial. For this reason, we also analysed the first session separately to examine whether the mirror/picture demonstration affected apes’ initial performance. The model including the three-way interaction between mirror experience, cue type, and demonstration was not significant (full–null model comparison: *χ*^2^ = 13.2, *df* = 7, *p* = 0.068), neither was the three-way interaction (*χ*^2^ = 0.02, *df* = 1, *p* = 0.902). A reduced model without the three-way interaction, however, was significant when compared to the null model (*χ*^2^ = 13.1, *df* = 6, *p* = 0.041). We found an interaction between mirror-experienced and demonstration that approached significance (*χ*^2^ = 3.73, *df* = 1, *p* = 0.053; see Fig. 8b). The interactions between mirror experience and cue type (*χ*^2^ = 0.01, *df* = 1, *p* = 0.909) or between cue type and demonstration (*χ*^2^ = 0.05, *df* = 1, *p* = 0.821) were clearly not significant.

When removing the interactions that did not approach significance (full–null model comparison: *χ*^2^ = 13.1, *df* = 4, *p* = 0.011; see Table S8), we found again that the interaction between mirror experience and demonstration approached significance (*χ*^2^ = 3.81, *df* = 1, *p* = 0.051). Subjects with mirror experience performed significantly better when they received the mirror demonstration compared to mirror-naïve individuals (*χ*^2^ = 7.96, *df* = 1, *p* = 0.005). In contrast, there was no difference between experienced and naïve individuals when they received the picture demonstration (*χ*^2^ = 0.08, *df* = 1, *p* = 0.775). Moreover, subjects overall selected the side with the food significantly more often when they were presented with mirrors than with pictures of mirror images (*χ*^2^ = 5.16, *df* = 1, *p* = 0.023).

Mirror-naïve apes performed overall better than chance when they received a mirror demonstration (mean ± SE: 0.63 ± 0.03; *T*^+^ = 36, *N* = 8, *p* = 0.008) but not when they received a picture demonstration (0.59 ± 0.05; *T*^+^ = 20, *N* = 6, *p* = 0.063). Experienced apes performed above chance with both the mirror (0.79 ± 0.07; *T*^+^ = 21, *N* = 6, *p* = 0.031) and picture demonstration (0.76 ± 0.07; *T*^+^ = 21, *N* = 6, *p* = 0.031).

### Discussion

The results of this experiment indicated that the mirror demonstration only affected the performance of mirror-experienced chimpanzees (i.e., individuals who had used mirror cues in Experiment 1) in their first session: these individuals performed significantly better than naïve individuals when they received the mirror demonstration. After a picture demonstration, there was no such difference between these groups. Overall, experienced individuals performed better with mirrors than with pictures (irrespective of the type of demonstration). Even though the picture of the mirror image was almost identical to the mirror image, at least with respect to its static properties, experienced individuals treated these cues differently.

A crucial difference between the mirror image and the picture were the dynamic aspects of a mirror image. Even though we tried to reduce subjects’ head movement using a juice dispenser, most of the time, apes were not completely still during trials. Especially, when *E* pushed the platform forward most apes stopped drinking, took their head back, and made their choice. In addition, the movement of the sliding platform changed the relative position of the mirrors to the apes. These relative movements between the mirrors and the subjects’ eyes might have increased the salience of the mirror images compared to the static pictures. Despite these differences between cues, we found that the type of demonstration affected the initial performance of mirror-experienced apes irrespective of the cue type when compared to naïve individuals. It is unclear whether this finding was driven by the mirror demonstration (e.g., serving as a reminder of the mirror properties) or by the picture demonstration (e.g., leading apes to discount the static, causally irrelevant cue). Finally, a note of caution is required here given the small sample size (and the resulting low power) of this between-subject analysis.

Mirror experience refers here to the competence apes have shown in the course of Experiment 1, 3, and 4. Mirror-naïve chimpanzees did not have any study-related mirror experience when they participated in the current experiment. Consistent with findings from mirror self-recognition studies, our results suggest that even limited experience with mirrors changes the way apes interpret mirror images. For example, Gallup (1970) reported that chimpanzees showed less social responses and more self-directed exploration toward their own mirror image after the second day of mirror exposure (with 8 h/day). Our findings support the notion that limited experience with mirrors allows chimpanzees to understand the relation between mirror reflection and its physical referent. However, we cannot exclude the possibility that some or all of the apes (including our mirror naïve individuals) had gained some prior experience with reflective surfaces (e.g., transparent glass surfaces or water surfaces that are part of their indoor and outdoor enclosures) before the study. Moreover, the mirror-experienced individuals were selected based on their performance in Experiment 1. This selection procedure might have contributed to the observed difference between mirror-experienced and naïve individuals.

## General discussion

The current set of experiments suggests that great apes are sensitive to certain optical relations in their environment. First, apes used cues of high similarity (mirror reflections) and modest similarity (shadows) with the food reward to localize the latter. Reinforcement learning seemed to be insufficient to account for apes’ performance given that the apes did not pick up equally deterministic and perceptually similar cues within the same experimental setup and number of trials. Second, apes pointed toward the real hiding place of a reflected food item instead of its mirror reflection suggesting that they could extract depth information from the mirror image. Third, apes showed some sensitivity to the orientation of a mirror to locate the reflected item. These latter two findings also ruled out that apes merely confused mirror reflections with their physical referents in terms of their spatial location. Finally, limited experience with mirrors led apes to interpret static pictures differently. Namely, mirror-experienced individuals tended to use a picture of a mirror image more readily when they expected to look at a mirror than when they received a demonstration indicating that the cue was a static picture. Taken together, these findings suggest that apes were sensitive to the correspondence between mirror reflections and shadows and their physical referents. This sensitivity to the involved optical relations seems to allow apes discriminate between appearance and reality in this context.

There are a number of limitations to the current results. In the present set of studies, we tested apes understanding of optical relations in a fairly constrained context involving only few mirror angles and their performance was not always very high. Using more interactive and dynamic setups akin to Menzel et al. (1985) might help in the future to further explore apes’ ability to make use of these optical relations. Furthermore, the two-choice setup with the subjects facing the experimenter always might be susceptible to inadvertent cueing (Pfungst 1965), However, we deem such Clever-Hans effects unlikely in this case for the following reasons: in the arbitrary control condition (Experiment 2), in which the experimenter gave cues to the apes by moving a rubber patch to the correct side of the platform, apes performed at chance. Any inadvertent cues that might have been given to the apes were most likely more subtle than the cues given in this arbitrary control condition. Moreover, apes modulated their pointing response depending on the distance of the food reward (Experiment 3). It is unclear how the experimenter would cue the apes to modulate their response accordingly (especially because we did not administer differential reinforcement for this response). Finally, in a pilot experiment with the same experimenter and a similar left/right choice apes failed to perform above chance. It is unclear why the apes would make use of any inadvertent cues in one setup but not the other.

Our findings are in line with a number of recent studies in which reinforcement learning failed to account for apes’ performance across various different paradigms (see Call 2006). In these studies, apes used, for example, the rattling noise of a food reward inside a shaken cup (Call 2004), the visual trail left by a leaky yogurt cup (Völter and Call 2014), or the orientation of a seesaw (Hanus and Call 2008) to locate hidden food. In some of these cases, apes performed above chance already in their first trial (Hanus and Call 2008; Völter and Call 2014) and often no significant improvement was found within the administered number of trials. All of these findings contrast with arbitrary control conditions in which there also was salient and reliable predictor of a food reward, which was not *causally* related to the presence of the reward. As in the arbitrary condition of Experiment 2 (in which the experimenter moved the rubber patch as a cue), apes largely failed to pick up these cues within an equal number of trials (Call 2004, 2007; Hanus and Call 2008, 2011; Völter and Call 2014; for a recent review, see Völter and Call 2017). Indeed, reinforcement learning can be surprisingly slow in apes if there is no apparent causal relation underpinning the reinforcement regime such as in token exchange paradigms (Hanus and Call 2011; Pelé et al. 2009; Schrauf and Call 2009). However, when the learning context is altered such that the tokens activate a food dispenser (thereby creating the impression of a causal relationship) instances of one-trial learning have been reported (Völter et al. 2016).

The current findings have implications for apes’ representational capacities. Using a novel paradigm we confirmed here that great apes are capable of appearance-reality discrimination in the context of mirror images. AR discrimination tasks typically require an individual to memorize the real task-relevant relations and to inhibit the misleading appearance information similar to Piagetian conservation tasks. Indeed, it has been argued that the nonverbal AR discrimination tasks that have been used to date cannot distinguish between AR discrimination and Piagetian conservation (Karg et al. 2014). Krachun et al. (2016), however, showed that even when visual tracking is prevented by shuffling the options out of sight, chimpanzees could use the misleading appearance to identify the truly larger food quantity. Mirrors offer additional possibilities to examine AR discrimination beyond conservation abilities. For example, if an individual is sensitive to the properties of a mirror, no pre-exposure of the real task relations would be necessary, which would rule out any Piagetian conservation account by design. In line with this, our results suggest that apes were indeed capable of discriminating between the mirror reflection and its referent without any pre-exposure of the actual food location. Indeed, apes’ performance suggest that they did not only discriminate between reflection and reflected object but they also seemed to infer the geometrical relation between the two.

Suddendorf and Whiten (2001) proposed that nonhuman great apes, like human children in the second year of life, hold secondary representations concurrently with primary perceptual representations. These secondary representations are decoupled from the perceptual input and might allow for the reinterpretation of primary representations (see also Karmiloff-Smith 1992). Evidence from diverse areas including mirror-induced self-exploration, hidden object displacement tasks, and means-end reasoning supports the claim that apes and young children entertain such secondary representations. Some of the most compelling evidence can be found in the literature on picture and scale model recognition. For instance, 2-year-old children can use pictures and scale models of a room to locate a hidden toy in the corresponding room after they had observed how an experimenter hid a miniature version of the toy in the analogous location of the model or picture (e.g., DeLoache 1991, 2000; DeLoache and Burns 1994). Likewise, chimpanzees have been shown to use a scale model of an enclosure to locate a hidden item after observing the hiding of a miniature version of the item in the corresponding position of the model (Kuhlmeier and Boysen 2001, 2002; Kuhlmeier et al. 1999). These findings suggest that young children and chimpanzees can use the correspondence relation between a model and its physical referent and they seem to use both relational and landmark cues to establish the correspondence between the model and its referent (DeLoache 1991; Kuhlmeier and Boysen 2002; Marzolf et al. 1999).

Our results provide further evidence that great apes entertain secondary representations. Primary perceptual representations of mirror reflections and shadows alone might lead to a misrepresentation of these optical effects. For example, a mirror reflection might be confused with its physical referent or it might be interpreted as a separate object without any relation to the referent. The current findings together with previous findings on mirror use in nonhuman primates (e.g., Anderson and Gallup 2015; Menzel et al. 1985) suggest that at least great apes do not misrepresent mirror reflections in this way. Secondary representations might allow apes to represent the correspondence between mirror reflections/shadows and their physical referents.

Finally, our results might have implications for self-recognition studies. As mentioned before, mirror-induced spontaneous self-exploration seems to be a capacity setting great apes apart from other primates (e.g., Anderson and Gallup 2015). Gorillas who sometimes seem to confuse pictures with real objects (Parron et al. 2008) are the only great apes species who produced mixed results with regard to self-recognition tests. A prerequisite for apes’ self-directed behaviours might be their causal understanding of mirror images as evidenced by the current findings. The only study that found self-directed mirror-induced behaviours in primates other than great apes used a training procedure to establish visual-somatosensory associations (Chang et al. 2015). After this training, rhesus macaques started to show self-exploratory behaviours such as touching marks on their face or exploring usually unseen body parts by means of a mirror. The reason why such training is not necessary for great apes might be that apes are capable of making inferences about the causes of optical effects such as their own mirror image. An interesting avenue for future research will be to extend the present work to other species, which might show whether the striking differences between great apes and other primates evident in self-recognition and picture recognition studies can be traced back to apes’ inferential reasoning capacity.

In summary, the current study shows that nonhuman great apes relate mirror images and shadows to objects in the world without confusing their location with the location of these objects. Control conditions suggest that reinforcement learning alone is insufficient to account for these findings. Moreover, apes showed themselves capable of locating hidden objects in space based on their mirror images. In particular, they could extract depth information from the mirror image and predicted the location of hidden food based on the orientation of the mirror. These inferences about optical relations may prove to be fundamental for apes’ outstanding performance in mirror-induced self-recognition tasks.

## Electronic supplementary material

Below is the link to the electronic supplementary material.

Supplementary material 1 (DOCX 3931 KB)

Supplementary material 2 (MP4 24063 KB)

Supplementary material 3 (MP4 19853 KB)

Supplementary material 4 (MP4 19939 KB)

Supplementary material 5 (MP4 32728 KB)

Supplementary material 6 (XLSX 318 KB)
