# Supplementary material for: Intuitive optics: what great apes infer from mirrors and shadows
Source: Anim Cogn. 2018 May 2;21(4):493–512. doi: 10.1007/s10071-018-1184-0 (PMC6004283; doi:10.1007/s10071-018-1184-0)

Title: **Intuitive Optics: What Great Apes Infer from Mirrors and Shadows**

Journal: Animal Cognition

Authors: Christoph J. Völter ^a,b*^**,** Josep Call ^a,b^

^a^ University of St Andrews, School of Psychology & Neuroscience, St Mary's Quad, South Street, St Andrews, Fife, KY16 9JP, Scotland, UK

^b^ Max Planck Institute for Evolutionary Anthropology, Department of Developmental and Comparative Psychology, Deutscher Platz 6, 04103 Leipzig, Germany

Corresponding Author: cjv3@st-andrews.ac.uk, Phone: + +44 (0)1334 46, ORCID iD: 0000-0002-8368-7201

**Supplementary Material**

# Table S1

*Experiment: 1 GLMM 01 output showing predictors of correct choices.*

| Term | Estimate | SE | 95% CI | | Χ² | DF | P |
| --- | --- | --- | --- | --- | --- | --- | --- |
| (Intercept) | 0.060 | 0.189 | -0.320 | 0.439 |  |  |  |
| Condition: control vs base^a^ | 2.013 | 0.227 | 1.608 | 2.470 | 38.655 | 1 | <0.001 |
| Condition: control vs mirror^a^ | 1.432 | 0.294 | 0.881 | 1.938 | 17.997 | 1 | <0.001 |
| Condition: control vs shadow^a^ | 1.078 | 0.226 | 0.650 | 1.549 | 17.113 | 1 | <0.001 |
| Condition: baseline vs mirror^d^ | -0.441 | 0.294 | -1.001 | 0.131 | 1.794 | 1 | 0.18 |
| Condition: baseline vs shadow^d^ | -0.782 | 0.234 | -1.227 | -0.343 | 8.081 | 1 | 0.004 |
| Condition: shadow vs mirror^d^ | 0.456 | 0.275 | -0.111 | 0.966 | 2.946 | 1 | 0.086 |
| Species: chimpanzee^b^ | -0.050 | 0.232 | -0.524 | 0.403 | 0.047 | 1 | 0.829 |
| Species: orangutan^b^ | 0.308 | 0.254 | -0.206 | 0.797 | 1.447 | 1 | 0.229 |
| Order | 0.026 | 0.112 | -0.197 | 0.265 | 0.054 | 1 | 0.816 |
| Sex^c^ | -0.272 | 0.207 | -0.686 | 0.155 | 1.680 | 1 | 0.195 |
| Age | 0.004 | 0.099 | -0.194 | 0.218 | 0.002 | 1 | 0.964 |
| Session | 0.102 | 0.104 | -0.113 | 0.312 | 0.956 | 1 | 0.328 |

*Note.* Reference categories: ^a^Condition: control, ^b^Species: bonobo, ^c^Sex: female; ^d^Post-hoc tests based on re-levelling of the reference category. All covariates were z-transformed. Confidence intervals (CI) were derived using the function bootMer of the R package lme4, using 1,000 parametric bootstraps and bootstrapping over the random effects, too.

# Table S2

Experiment 2: GLMM 02 output showing predictors of correct choices.

| Term | Estimate | SE | 95% CI | | Χ² | DF | P |
| --- | --- | --- | --- | --- | --- | --- | --- |
| (Intercept) | 0.484 | 0.243 | 0.013 | 0.997 |  |  |  |
| Condition: arbitrary vs baseline^a^ | 1.830 | 0.301 | 1.307 | 2.476 | 18.528 | 1 | <0.001 |
| Condition: arbitrary vs control^a^ | -0.080 | 0.184 | -0.439 | 0.262 | 0.188 | 1 | 0.664 |
| Condition: arbitrary vs shadow^a^ | 0.640 | 0.258 | 0.172 | 1.175 | 5.322 | 1 | 0.021 |
| Condition: control vs baseline^c^ | 1.910 | 0.301 | 1.376 | 2.566 | 19.175 | 1 | <0.001 |
| Condition: control vs shadow^c^ | 0.719 | 0.257 | 0.176 | 1.261 | 6.519 | 1 | 0.011 |
| Condition: shadow vs baseline^c^ | 1.220 | 0.298 | 0.648 | 1.852 | 13.155 | 1 | <0.001 |
| Order | -0.187 | 0.153 | -0.478 | 0.129 | 1.497 | 1 | 0.221 |
| Sex^b^ | -0.094 | 0.174 | -0.455 | 0.252 | 0.294 | 1 | 0.588 |
| Age | -0.013 | 0.007 | -0.028 | 0.001 | 3.189 | 1 | 0.074 |
| Session | 0.128 | 0.148 | -0.182 | 0.439 | 0.747 | 1 | 0.387 |

*Note.* Reference categories: ^a^Condition: arbitrary, ^b^Sex: female; ^c^Post-hoc tests based on re-levelling of the reference category. All covariates were z-transformed. Confidence intervals (CI) were derived using the function bootMer of the R package lme4, using 1,000 parametric bootstraps and bootstrapping over the random effects, too.

# Table S3

Experiment 3: GLMM 03 output showing predictors that influenced subjects’ choice of the correct side.

| Term | Estimate | SE | 95% CI | | Χ² | DF | P |
| --- | --- | --- | --- | --- | --- | --- | --- |
| (Intercept) | 5.088 | 1.142 | 3.526 | 9.863 |  |  |  |
| Visibility^a^ | -3.926 | 1.044 | -8.632 | -2.536 | 24.149 | 1 | <0.001 |
| Distance^b^ | 1.794 | 0.435 | 1.134 | 3.547 | 16.286 | 1 | <0.001 |
| Session | 0.392 | 0.816 | -1.194 | 2.079 | 0.232 | 1 | 0.630 |
| Species: chimpanzee^c^ | 0.134 | 0.692 | -1.513 | 1.441 | 0.037 | 1 | 0.847 |
| Species: orangutan^c^ | 0.366 | 0.743 | -1.244 | 1.917 | 0.239 | 1 | 0.625 |
| Trial | -0.256 | 0.783 | -1.939 | 1.302 | 0.107 | 1 | 0.744 |

*Note.* Reference category: ^a^Visibility: clear, ^b^Distance: close, ^c^Species: bonobo. All covariates were z-transformed. Confidence intervals (CI) were derived using the function bootMer of the R package lme4, using 1,000 parametric bootstraps and bootstrapping over the random effects, too.

# Table S4

Experiment 3: GLMM 04 output showing predictors that influenced subjects’ probability to point to the proximal screen.

| Term | Estimate | SE | 95% CI | | Χ² | DF | P |
| --- | --- | --- | --- | --- | --- | --- | --- |
| (Intercept) | 3.660 | 1.172 | 1.540 | 6.297 |  |  |  |
| Visibility^a^ | -0.135 | 0.594 | -1.332 | 1.125 |  |  |  |
| Distance^b^ | -3.446 | 0.462 | -4.510 | -2.564 |  |  |  |
| Session | 0.251 | 0.215 | -0.169 | 0.675 | 1.337 | 1 | 0.248 |
| Species: chimpanzee^c^ | -1.202 | 1.297 | -4.267 | 1.294 | 0.830 | 1 | 0.362 |
| Species: orangutan^c^ | -1.182 | 1.362 | -4.235 | 1.471 | 0.735 | 1 | 0.391 |
| Visibility^a^ : Distance^b^ | 1.435 | 0.592 | 0.213 | 2.675 | 5.661 | 1 | 0.017 |

*Note.* Reference category: ^a^Visibility: clear, ^b^Distance: close, ^c^Species: bonobo. The covariate Session was z-transformed. Confidence intervals (CI) were derived using the function bootMer of the R package lme4, using 1,000 parametric bootstraps and bootstrapping over the random effects, too.

# Table S5

Experiment 4: GLMM 05 output showing predictors that influenced whether subjects pointed to the side where they could see the mirror image or picture of the food.

| Term | Estimate | SE | 95% CI | | Χ² | DF | P |
| --- | --- | --- | --- | --- | --- | --- | --- |
| (Intercept) | 1.164 | 0.345 | 0.511 | 1.886 |  |  |  |
| Congruence^a^ | -0.329 | 0.187 | -0.690 | 0.047 |  |  |  |
| Cue type^b^ | -0.923 | 0.281 | -1.474 | -0.421 |  |  |  |
| Order of cue type | -0.188 | 0.288 | -0.758 | 0.439 |  |  |  |
| Species: chimpanzee^c^ | -0.075 | 0.237 | -0.599 | 0.420 | 0.103 | 1 | 0.748 |
| Species: orangutan^c^ | -0.422 | 0.310 | -0.991 | 0.160 | 2.143 | 1 | 0.143 |
| Congruence^a^ : Cue type^b^ | 0.277 | 0.249 | -0.212 | 0.757 |  |  |  |
| Congruence^a^ : Order | 0.570 | 0.187 | 0.193 | 0.945 |  |  |  |
| Cue type^b^ : Order | 0.108 | 0.355 | -0.650 | 0.788 |  |  |  |
| Congruence^a^ : Cue type^b^ : Order | -0.738 | 0.249 | -1.247 | -0.245 | 8.741 | 1 | 0.003 |

*Note.* Reference category: ^a^Congruence: congruent, ^b^Cue type: mirror, ^c^Species: bonobo. All covariates were z-transformed. Confidence intervals (CI) were derived using the function bootMer of the R package lme4, using 1,000 parametric bootstraps and bootstrapping over the random effects, too.

# Table S6

Experiment 4: GLMM 06 output showing predictors that influenced whether subjects pointed to the side where they could see the mirror image or picture of the food in session 1.

| Term | Estimate | SE | 95% CI | | Χ² | DF | P |
| --- | --- | --- | --- | --- | --- | --- | --- |
| (Intercept) | 0.319 | 0.442 | -0.529 | 1.274 |  |  |  |
| Congruence^a^ | -0.670 | 0.479 | -1.785 | 0.295 |  |  |  |
| Cue type^b^ | -0.444 | 0.569 | -1.648 | 0.705 |  |  |  |
| Species: chimpanzee^c^ | 1.460 | 0.459 | 0.688 | 2.592 | 8.801 | 1 | 0.003 |
| Species: orangutan^c^ | 0.681 | 0.489 | -0.327 | 1.734 | 1.952 | 1 | 0.162 |
| Congruence^a^ : Cue type^b^ | 0.985 | 0.739 | -0.562 | 2.527 | 1.792 | 1 | 0.181 |

*Note.* Reference category: ^a^Congruence: congruent, ^b^Cue type: mirror, ^c^Species: bonobo. Confidence intervals (CI) were derived using the function bootMer of the R package lme4, using 1,000 parametric bootstraps and bootstrapping over the random effects, too.

# Table S7

Experiment 5: GLMM 07 output showing predictors that influenced whether subjects pointed to the side where they could see the mirror image or picture of the food.

| Term | Estimate | SE | 95% CI | | Χ² | DF | P |
| --- | --- | --- | --- | --- | --- | --- | --- |
| (Intercept) | 1.994 | 0.402 | 1.298 | 2.888 |  |  |  |
| Cue type^a^ | -0.975 | 0.356 | -1.801 | -0.267 |  |  |  |
| Mirror experience^b^ | -1.445 | 0.479 | -2.439 | -0.598 |  |  |  |
| Demonstration^c^ | -0.178 | 0.366 | -0.889 | 0.617 |  |  |  |
| Order of demonstration | 0.083 | 0.087 | -0.115 | 0.278 | 0.903 | 1 | 0.342 |
| Cue type^a^ : Mirror experience^b^ | 1.007 | 0.373 | 0.252 | 1.848 | 7.425 | 1 | 0.006 |
| Cue type^a^ : Demonstration^c^ | 0.019 | 0.350 | -0.680 | 0.747 | 0.003 | 1 | 0.956 |
| Mirror experience^b^ : Demonstration^c^ | 0.003 | 0.368 | -0.776 | 0.787 | 0.000 | 1 | 0.994 |

*Note.* Reference category: ^a^Cue type: mirror, ^b^Mirror experience: experienced, ^c^Demonstration: mirror. The covariate Order of demonstration was z-transformed. Confidence intervals (CI) were derived using the function bootMer of the R package lme4, using 1,000 parametric bootstraps and bootstrapping over the random effects, too.

# Table S8

Experiment 5: GLMM 08 output showing predictors that influenced whether subjects pointed to the side where they could see the mirror image or picture of the food in session 1.

| Term | Estimate | SE | 95% CI | | Χ² | DF | P |
| --- | --- | --- | --- | --- | --- | --- | --- |
| (Intercept) | 2.076 | 0.525 | 1.192 | 3.448 |  |  |  |
| Cue type^a^ | -0.765 | 0.341 | -1.436 | -0.122 | 5.162 | 1 | 0.023 |
| Mirror experience^b^ | -1.694 | 0.571 | -3.090 | -0.680 |  |  |  |
| Demonstration^c^ | -1.096 | 0.604 | -2.561 | 0.093 |  |  |  |
| Mirror experience^b^ : Demonstration^c^ | 1.539 | 0.755 | 0.153 | 3.269 | 3.807 | 1 | 0.051 |

*Note.* Reference category: ^a^Cue type: mirror, ^b^Mirror experience: experienced, ^c^Demonstration: mirror. Confidence intervals (CI) were derived using the function bootMer of the R package lme4, using 1,000 parametric bootstraps and bootstrapping over the random effects, too.

# **Fig. S1**

Experiment 1 and 2: Photographs showing experimental setup from the subjects’ perspective. a) Shadow condition, b) Mirror condition. The platforms are shown right before subjects could choose by inserting their hands into one of the response windows in the panel.


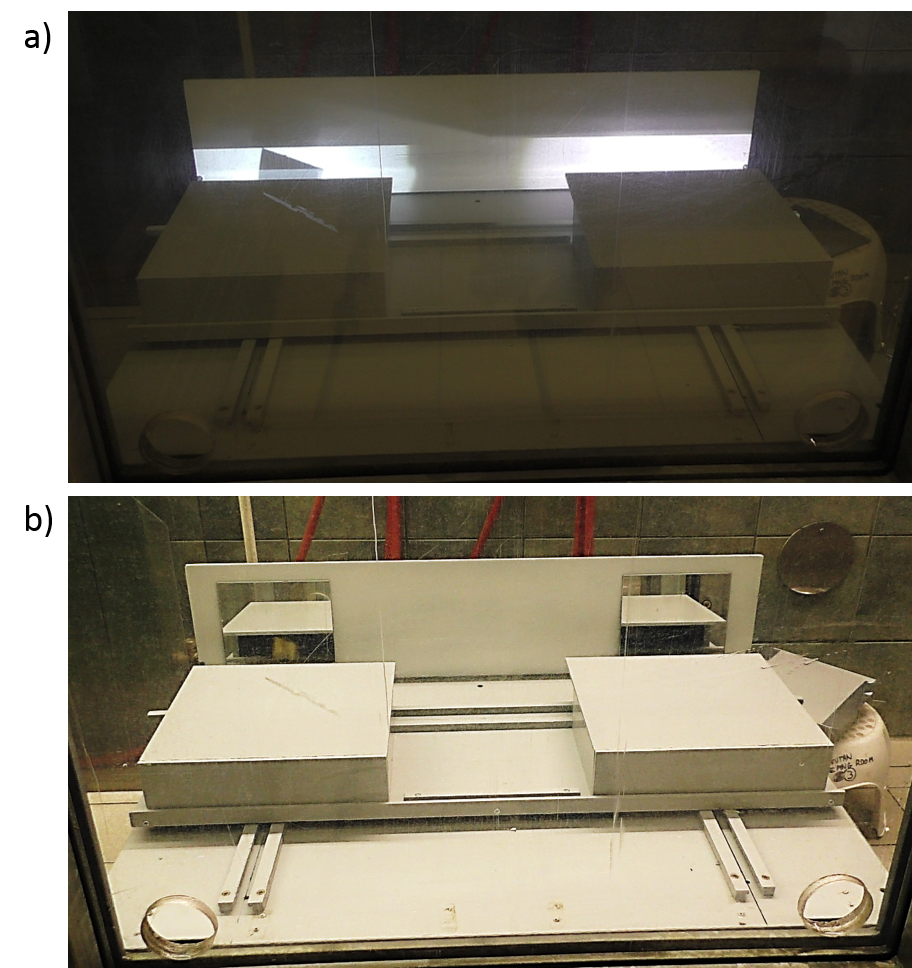


# **Fig. S2**

Experiment 3: Photograph showing experimental setup. The subject sat behind the mesh panel on the right.


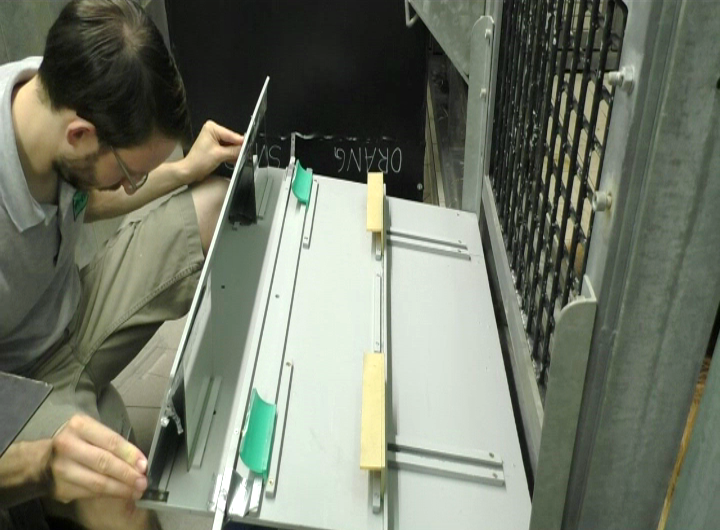


# **Fig. S3**

Experiment 4: Photographs showing the mirror images from the subjects’ perspective. a) Incongruent condition; b) Congruent condition.


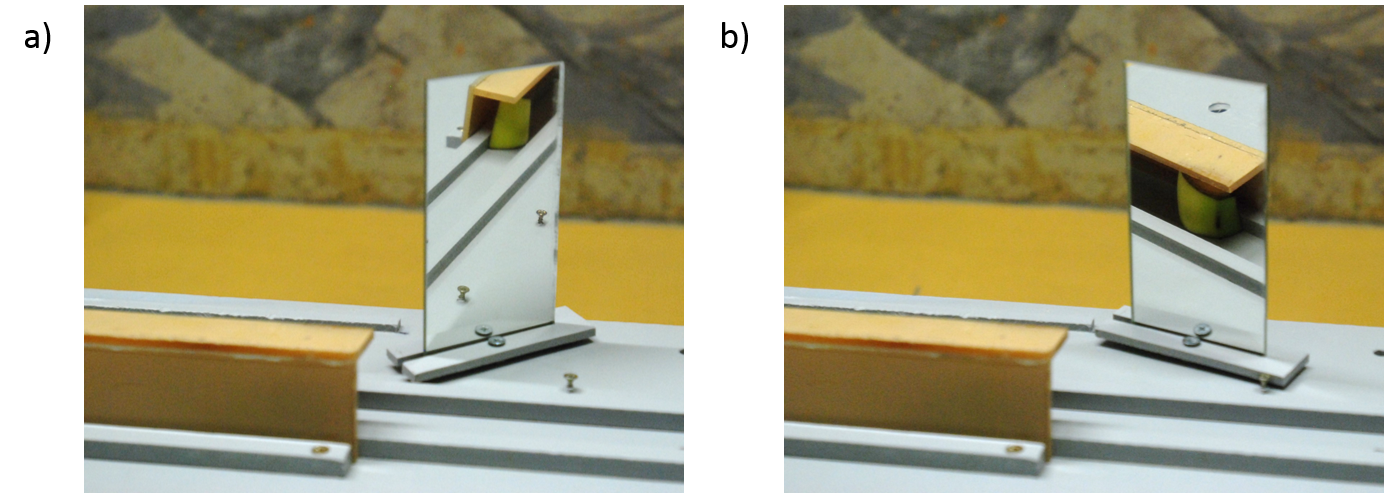


# **Fig. S4**

Experiment 5: Example of the experimental stimuli. For illustration purposes, pictures and mirrors are presented here together on the sliding platform. In the experiment, subjects could never directly compare pictures and mirror images. Moreover, in the actual experiment, the banana was visible only on one side; on the non-baited side, there was a picture / mirror image showing the same background without the banana. Pictures were taken from the perspective of the apes when they were drinking juice through the hole in the panel. a) left: mirror, right: picture; b) left: picture, right: mirror.


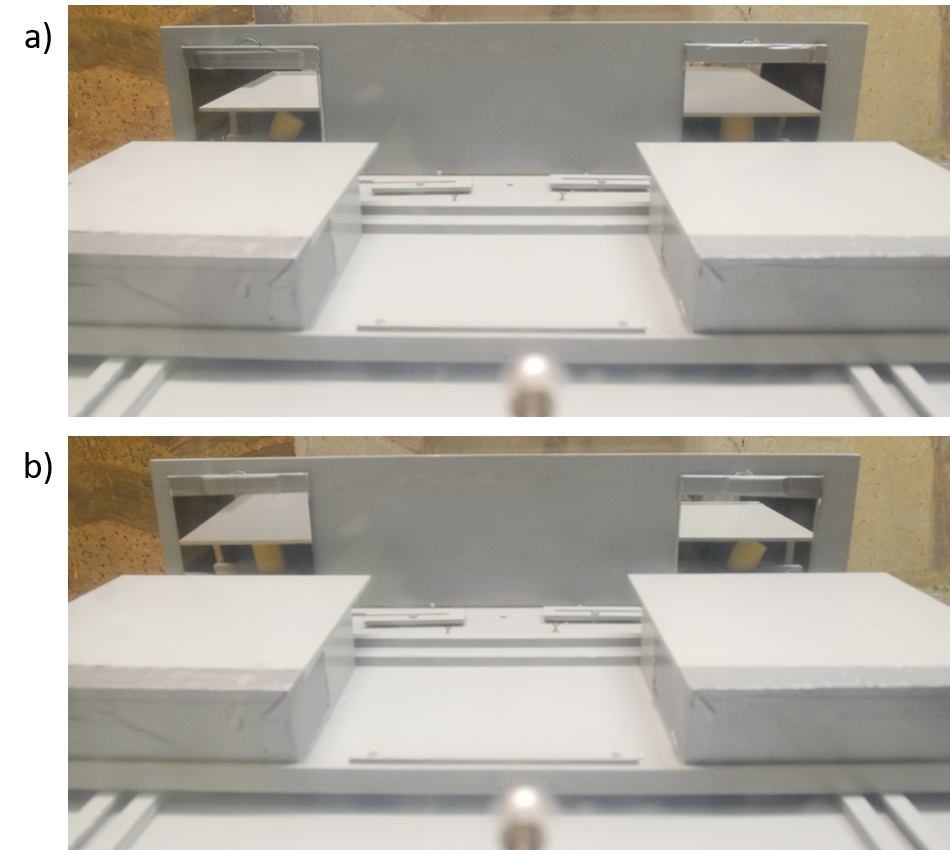

Supplement: Supplementary file 1 — Supplementary material 1 (DOCX 3931 KB) [file 10071_2018_1184_MOESM1_ESM.docx]
